# Age and Alzheimer’s disease affect functional connectivity along separate axes of functional brain organization

**DOI:** 10.1101/2025.05.22.655469

**Published:** 2025-05-25

**Authors:** Jonathan Rittmo, Nicolai Franzmeier, Olof Strandberg, Léa Chauveau, Theodore D. Satterthwaite, Laura EM Wisse, Nicola Spotorno, Harry H Behjat, Amir Dehsarvi, Danielle van Westen, Toomas Erik Anijärv, Sebastian Palmqvist, Shorena Janelidze, Erik Stomrud, Rik Ossenkoppele, Niklas Mattsson-Carlgren, Oskar Hansson, Jacob W Vogel

**Affiliations:** 1 Department of Clinical Sciences Malmö, Faculty of Medicine, SciLifeLab, Lund University, Lund, Sweden; 2 Institute for Stroke and Dementia Research (ISD), University Hospital, LMU Munich, 81377 Munich, Germany; 3 Munich Cluster for Systems Neurology (SyNergy), 81377 Munich, Germany; 4 Department of Psychiatry and Neurochemistry, Institute of Neuroscience and Physiology, Sahlgrenska Academy, University of Gothenburg, 405 30 Mölndal and Gothenburg, Sweden; 5 Clinical Memory Research Unit, Department of Clinical Sciences Malmö, Faculty of Medicine, Lund University, Lund, Sweden; 6 Penn-Children’s Hospital of Philadelphia Lifespan Brain Institute, University of Pennsylvania, Philadelphia, PA 19104; 7 Data Science for Integrated Diagnostics (AI2D), Perelman School of Medicine, University of Pennsylvania, Philadelphia, PA, USA; 8 Penn Lifespan Informatics and Neuroimaging Center (PennLINC), Philadelphia, PA, United States; 9 Department of Psychiatry, University of Pennsylvania Perelman School of Medicine, Philadelphia, PA, United States; 10 Department of Diagnostic Radiology, Department of Clinical Sciences Lund, Lund University, Lund, Sweden; 11 Memory Clinic, Skåne University Hospital, SE-205 02 Malmö, Sweden; 12 Alzheimer Center Amsterdam, Neurology, Vrije Universiteit Amsterdam, Amsterdam UMC, Amsterdam, the Netherlands; 13 Amsterdam Neuroscience, Neurodegeneration, Amsterdam, the Netherlands

## Abstract

Aging and Alzheimer’s disease (AD) are associated with alterations in functional connectivity (FC), yet their spatial and temporal characteristics remain debated. Whole-cortex functional gradients, which organize regions along axes of functional similarity, are positioned here as a framework for understanding such alterations. Across two independent cohorts (BioFINDER-2, N=973; ADNI, N=129), we demonstrate that hyper- and hypoconnectivity in both healthy aging and biomarker-confirmed AD progression coexist systematically, forming consistent spatial patterns that align with two distinct axes of brain organization. Using a combination of longitudinal and non-linear analyses, we show that the early (but not late) stages of AD pathology accumulation is associated with functional alteration along the sensory-association axis, a pattern that vanishes in later stages of AD. However, functional alteration along the sensory-association axis is associated with worse cognition throughout the AD spectrum, and even in older adults without AD pathology, suggesting that these FC patterns may reflect a general neural response to cognitive strain. Independently of AD, older age was associated with alterations instead along the executive-nonexecutive axis, a finding that was consistent throughout the adult lifespan. These findings highlight the fundamental role of intrinsic functional organization in shaping how the brain responds to aging and to AD, helping to resolve previously reported discrepancies.

Aging and Alzheimer’s disease (AD) are both associated with changes in functional brain networks – groups of regions that typically communicate with one another and support cognition. However, the precise nature of these changes – their spatio-temporal dynamics, and whether they reflect compensatory adaptations, pathological disruptions, or both – remains a topic of active debate^1,2^. Answering these questions is not only important for understanding disease biology, but could enable tailored clinical strategies that address age-independent mechanisms of AD. AD is the leading cause of dementia and is characterized by pathological accumulation of amyloid-β and tau proteins. These proteins spread through the brain in characteristic patterns^3,4^, leading to neurodegeneration and cognitive decline. Functional connectivity (FC), the temporal synchrony of activity between brain regions, may be a mechanism facilitating the spread of pathology, a system affected by pathology, or an interplay of both^5–8^. However, normal or healthy aging is also associated with altered functional connectivity^9–15^. These age and AD-related alterations have been proposed both as mechanisms to sustain cognitive function as the brain is challenged by stressors, and as manifestations of pathological network breakdown, e.g.^2,6,16–20^.

FC changes in aging and AD have commonly been studied through two approaches. One approach focuses on regional connectivity changes, such as increases or decreases within or between specific regions or networks, or between regions of interest and the rest of the brain^21–24^. Although some consistent effects have been reported, such as reduced FC within the default mode network in healthy aging^10^, the overall picture is more complex. Studies of aging, especially in the context of AD, frequently report mixed patterns of hyper- and hypoconnectivity, among many:^25–34^. Such mixed findings may arise from several sources. Contributing factors include the predominantly cross-sectional nature of existing studies, unmeasured pathology, region- or network-specific analytical approaches that may miss broader spatial patterns, and small sample sizes that may not represent the broader population. An additional possibility is that the relationship between connectivity and age or disease progression is nonlinear – that is, hyper- may shift to hypoconnectivity (or vice versa) depending on age or disease severity. While initial hyperconnectivity is often attributed to adaptive cognitive compensation^2,6^, the relationship between connectivity and cognition remains unclear. The common regional approach of FC research allows for precise and granular analyses, but is inherently limited in providing a more holistic perspective on functional changes in healthy and (AD) pathological aging.

The other common approach for studying FC changes in aging and AD adopts a more holistic view, analyzing connectivity patterns by decomposing connectivity matrices into components of variance often referred to as ‘gradients’^35^. These components organize brain regions along axes of functional similarity, representing whole-cortex patterns of brain organization. The primary components capture the largest variations in the data, arranging regions along a continuum that groups areas with similar brain activity together, while placing regions with the greatest dissimilarities at opposite ends. For example, the sensory-association axis^36^, which is often the primary variance component of FC^35,37^, groups regions involved in sensory and motor function (unimodal) on one end of the gradient spectrum, and those supporting higher-order associative cognition (transmodal) on the other. Typically, the second component spans the visual and motor cortices, while the third component groups executive networks at one end and all other networks at the opposite. A main feature of the gradients lies in capturing the brain’s functional architecture as continuous, overlapping maps ranking regions by their degree of involvement in distinct functional domains. But, the typical way to study gradients is in terms of how their expression changes, e.g. they become more pronounced during development^38–40^ and less pronounced in age or with AD^7,41,42^.

In this study, we do not use functional gradients as the object of study, distinguishing us from the aforementioned prior literature. Instead, we use functional gradients as a framework to interpret the spatial distribution of connectivity changes in aging and AD. By doing so, we integrate the two dominant approaches outlined above, combining regional granularity with a whole-cortex functional perspective. However, capturing a comprehensive picture of how FC is altered by aging and AD requires consideration of not only where these changes occur, but also when. To address this, we model nonlinear region-wise trajectories of connectivity across age and along the AD continuum using a continuous biomarker-based measure of pathology, then examine how longitudinal increases in pathology affect concurrent changes in connectivity. Finally, to determine whether these connectivity changes relate to cognition independently of age and pathology, we conduct separate analyses in cognitively unimpaired and cognitively impaired individuals. Altogether, this study provides a framework that leverages group-level functional gradients and a continuous biomarker-based pathology measure to examine the spatial distribution, cognitive relevance, and temporal progression of FC changes in aging and AD.

## Results

**Figure 1:**
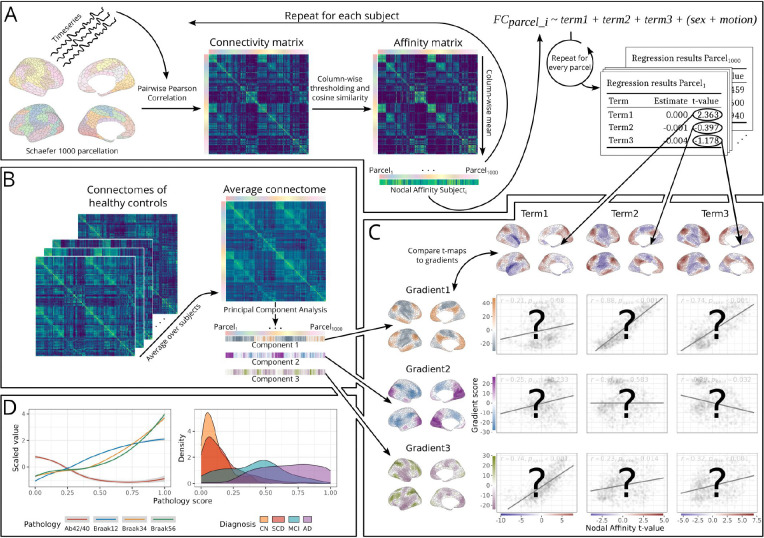
Mapping functional connectivity changes to cortical gradients. (A) Resting-state fMRI data for each subject were parcellated into 1000 regions^43^. Timeseries from these parcels underwent pairwise Pearson correlation. Cosine pairwise similarity between the connectivity profiles of the parcels were then calculated and averaged for each parcel, resulting in one FC value per parcel and subject^35^. This served as the outcome in parcelwise regression models, with t-values of the terms of interest (e.g., age, diagnosis) mapped onto the cortical surface in (C), forming t-maps. (B) Gradients were derived by averaging connectivity matrices of healthy controls and calculating principal components. The first three components/gradients were retained but only the first and third further analysed. (C) T-maps are compared to gradients and their relationship quantified using spin tests. (D) Participants were given a pathology score by mapping them nonlinearly onto a continuous pathology trajectory using Aβ and tau biomarkers using the SCORPIUS method^45^, providing a gradated and biological alternative to clinical diagnoses for subsequent analyses (see Methods for details). The relationship (LOESS curves) between the pathology score and the scaled biomarkers used to derive it is shown to the left, and distributions of clinical diagnosis groups to the right.

We analyzed resting-state fMRI data from the BioFINDER-2 cohort (973 participants) with complete data (CSF Aβ42/40, tau PET and fMRI scans; see Table 1 for participant characteristics). After parcellating the cortex into 1000 regions^43^, FC was quantified using nodal affinity, the parcel-wise average of connectivity similarity to all other parcels (see Figure 1 A and Methods). Nodal affinity was used as the outcome in parcel-wise regression models (see Methods), with the t-values of the terms of interest projected onto the cortical surface, resulting in “t-maps” (Figure 1 A, C). Note that very similar results were found using nodal connectivity strength as outcome (see Figure S1). Our goal was to assess the degree to which these t-maps aligned with the principal axes (i.e. gradients) of functional organization (Figure 1 C). The gradients of interest were defined as the first three principal components of the average connectomes from healthy controls (Figure 1 B; see Figure S2 for comparisons of different methods to derive gradients, and explained variance of the derived components). Supplementary analyses (Table S1) with gradients derived using several methods (including the traditional diffusion map embedding from^35^ confirmed that this methodological choice did not substantially impact the results. The age- and AD-related t-maps were spatially compared to the first three gradients, using Pearson correlation to quantify their relationship with statistical significance assessed using a spin test to account for spatial autocorrelation^44^.

In studies comparing aging and AD it is common to rely on clinical diagnostic criteria. However, these criteria may confound pathological processes with cognitive factors (e.g., resilience). To isolate pathological AD effects, we used a continuous measure of pathology severity by nonlinearly mapping participants onto a continuous trajectory based on CSF Aβ42/40 ratio and tau PET using the SCORPIUS method^45^. This continuous pathology measure was used instead of clinical diagnoses to quantify AD disease progression in all subsequent analyses (but see Figure S3 for an analysis with clinical diagnoses). In Figure 1 D the relationship between this composite pathology score and its constituents is visualized, along with its distribution over clinical groups.

### Cross-sectional effects of age and AD pathology on functional connectivity align with distinct gradients

**Figure 2:**
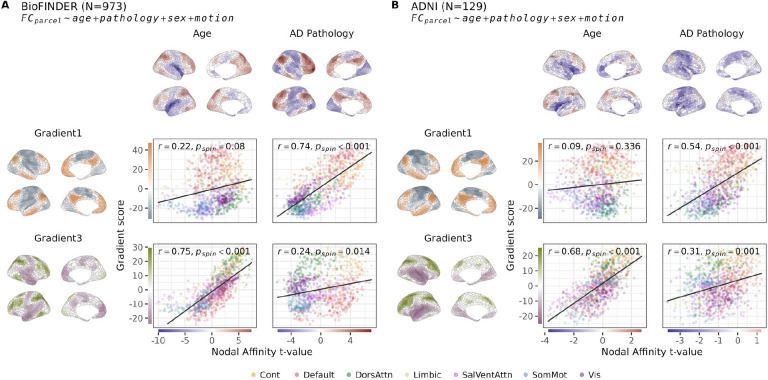
Age and AD pathology are associated with functional connectivity changes aligned with distinct organizational gradients. These dissociable alignments were observed in BioFINDER-2 (A), and in an external cohort, ADNI (B). Cortical maps display t-values from nodal linear regression models with nodal affinity as the outcome, using age and Alzheimer’s pathology as independent variables, covaried for sex and motion. Scatter plots show the relationship between t-values and gradient scores across nodes, colored by network membership. The relationship was quantified using Pearson correlation and significance assessed using a spin test. Color scales on the axes match the corresponding cortical t-maps. See Figure S4 for network delineation of the cortical maps of A.

In the BioFINDER-2 cohort, the spatial distribution of independent nodal associations between FC and AD pathology (i.e. Figure 1 D) aligned with Gradient 1 (r = 0.74, p_spin_<0.001), reflecting decreases of FC in sensory-motor (unimodal) regions and increases in associative (transmodal) regions with increasing pathology. Age effects were instead independently aligned with Gradient 3 (r = 0.75, p_spin_<0.001), with executive areas showing increasing and non-executive areas decreasing FC with increasing age (Figure 2 A, Figure S4). Notably, age effects were not strongly aligned with Gradient 1, and AD effects were not strongly aligned with Gradient 3, suggesting a double dissociation. These findings were replicated using a subset of the ADNI cohort (129 participants with complete data), see Figure 2 B (Gradient 1 for pathology: r = 0.54, p_spin_<0.001; Gradient 3 for age: r = 0.68, p_spin_<0.001). Due to a non-existant or modest and non-reproducible relationship between the t-maps and Gradient 2 (see Figure S5), this gradient was left out of the present and subsequent analyses for brevity. Separate analyses of within- and between-network affinity (Figure S6, Figure S7) revealed similar patterns to overall affinity, with generally weaker gradient relationships for within- compared to between-network affinity. This suggests that both contribute to the observed gradient alignments, but between-network connections play a greater role.

### FC effects and their alignment with gradients vary along the age- and pathology spectra

**Figure 3:**
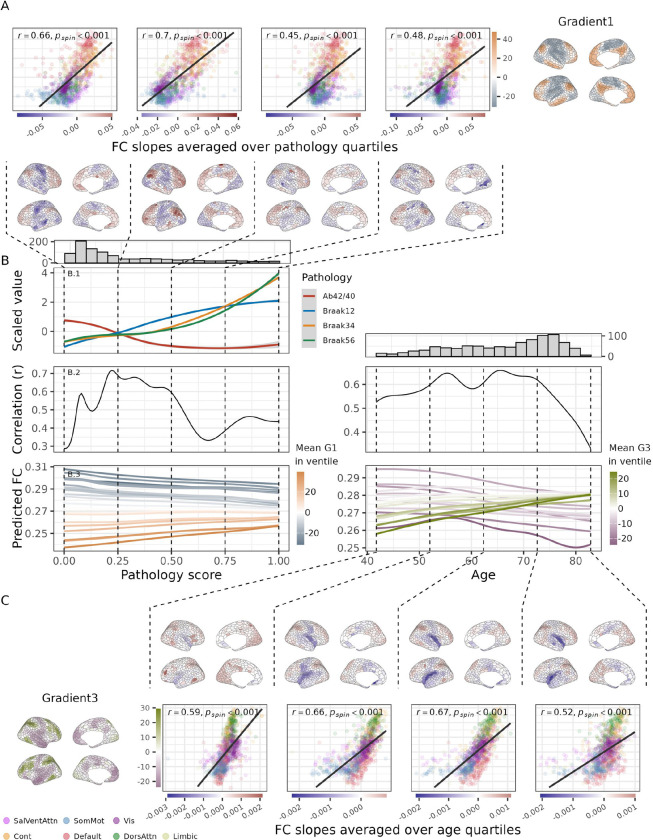
Nonlinear models reveal that the alignment of age- and pathology-related FC effects with organizational gradients varies across the age and AD pathology continuum. Generalized additive models (GAMs) were fitted for each parcel, with nodal affinity as the outcome, and age and AD pathology as (non-linear) smooth terms, while controlling for sex and motion. Predicted trajectories and their derivatives were calculated. (A): Top row: Scatter plots of averaged slope coefficients across pathology quartiles vs. Gradient 1 scores, with parcels colored by network membership. Bottom row: Cortical maps of averaged slope coefficients. (B.1): nonlinear relationship between the composite AD pathology score and scaled pathology measures. (B.2): Correlation coefficients showing how alignment between trajectory derivatives and gradient scores changes across pathology (Gradient 1) and age (Gradient 3). (B.3): Predicted nodal affinity trajectories for pathology and age, averaged within gradient-based ventiles (20 equally sized groups), colored by mean gradient scores. Marginal histograms in B shows the sample sizes across pathology score and age from the original estimation. (C): Same as A, but for age quartiles.

Prior studies on FC in AD have reported an initial pattern of regional hyperconnectivity, which is subsequently followed by connectivity declines^1,34,46^. Nonlinear relationships between age and FC have also been reported, e.g.^15,31^. We therefore sought to investigate whether these nonlinearities were reflected similarly in gradient-like FC changes. To investigate these potential nonlinearities, generalized additive models (GAMs) were fitted with FC at each parcel as the outcome, and age, pathology and relevant covariates as predictors. The derivatives of these models capture the direction and strength (i.e., slope) of each predictor’s effect across its range. For each predictor (age and pathology), the estimated slopes result in cortical “slope-maps” that reflect the regional strength and direction of the predictor’s effect, at any value of the predictor. This allows us to observe how FC increases or decreases at specific points across the age and pathology spectra. For pathology, the relationship between Gradient 1 and the slope-maps increased up to a pathology level of ~0.25 (representing early AD pathology), remaining relatively high until ~0.5 (representing middle-stages of AD pathology), after which it declined sharply with further accumulation (Figure 3 B.2). For age, the relationship between Gradient 3 and the slope-maps remained high between ages 55 and 70, but essentially vanished after age 80. These results suggest that gradient-like FC patterns emerge as an initial response to pathological or physiological changes but diminish as these changes progress.

Regional FC trajectories, averaged within 20 quantile bins (ventiles) based on gradient values, are shown in Figure 3 B.3. Across the pathology spectrum, average FC in transmodal ventiles increased relatively linearly, while FC in unimodal ventiles decreased at a similar rate. Sensory-motor (unimodal) ventiles consistently exhibited higher FC than associative (transmodal). Across age, FC increased in executive ventiles and decreased in non-executive ventiles. While executive ventiles had lower FC than non-executive regions in younger age, this pattern reversed in older age.

### Longitudinal increase in pathology is associated with gradient-aligned connectivity changes

**Figure 4:**
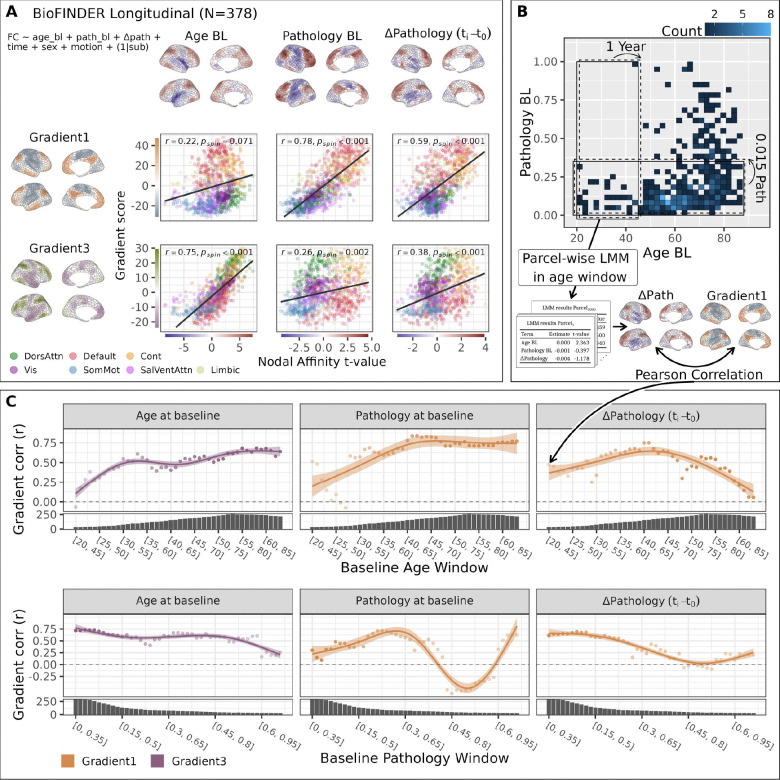
Within-subject change in pathology is related to gradient-aligned connectivity effects. A: A linear mixed-effects model was applied at each parcel, with FC as the outcome and baseline age, baseline pathology (tau PET SUVR from Braak I-VI), Δ pathology (longitudinal change in tau PET from baseline) as fixed effects and random intercepts for subjects, covaried for time since baseline, sex and scanner motion. Cortical maps display t-values from the parcel-wise models, while scatter plots show the relationship between t-values and gradient scores, colored by network membership. The relationship was quantified using Pearson correlation and significance assessed using a spin test. Color scales of the cortical maps are shown along the axes of the scatter plots. B: Schematic of the sliding window approach used to assess longitudinal nonlinearities in the relationship between FC and gradients across the aging and pathology spectra. The heatmap represents a bivariate histogram; the color of each tile indicates the number of subjects in each histogram bin. Windows are represented as rectangles “moving” across the bivariate histogram. Age windows spanned 25 years and were incremented by 1 year, while pathology windows spanned 0.35 pathology score units and were incremented by 0.015, resulting in 44 age windows and 45 pathology windows, all having ≥ 25 subjects. For each window we fit linear mixed models at the parcel level, t-maps for each fixed effect of interest were extracted and the t-maps correlated (Pearson correlation) with Gradient 1 and 3. C: Results of the sliding window analysis showing the dynamic relationship of AD pathology on FC gradient alignment. The correlation values on the scatters are overlaid with an estimation from generalized additive models. For the age terms in each windowing, correlations are shown between the t-map and Gradient 3, while for the pathology terms, they are shown between the t-maps and Gradient 1. Marginal plots show the sample size for each window. Results from the age windowing show that baseline age and pathology effects on Gradients 3 and 1, respectively, are mostly stable across the adult lifespan. Across the pathology windows, baseline pathology effects increase in gradient alignment over the initial windows after which they decrease. This pattern was mirrored in Δ pathology, but the within subject change preceded the baseline effects with peaks and declines in earlier windows.

A subset of participants in the BioFINDER-2 sample (N = 378) had longitudinal data available for all variables except CSF Aβ (see Table 2 for longitudinal characteristics, number of visits etc.). To determine whether the gradient-aligned FC changes from previous analyses reflect within-subject responses to pathology (as opposed to cross-sectional associations), we fit linear mixed-effects models. Since longitudinal CSF Aβ data were unavailable, the pathology score was re-calculated using only tau PET from Braak I-II, III-IV and V-VI regions of interest. The baseline effects in this sample (representing between subject differences; Figure 4 A) closely mirrored the previous cross-sectional findings (Figure 4 A). The longitudinal change in pathology (ΔPathology, representing within-subject changes) also showed gradient-aligned connectivity changes (G1: r = 0.59, p_spin_<0.001), confirming that within-subject pathology accumulation is associated with within-subject FC increases and decreases along organisational axes. Interestingly, there was also a modest relationship between Gradient 3 (r = 0.38, p_spin_<0.001) and longitudinal change in pathology.

To explore whether the previously identified nonlinearities were also reflected longitudinally, we employed a sliding window approach, examining how gradient alignment varies across different baseline ages and baseline pathology levels. Figure 4 B provides a methodological overview, and Figure 4 C displays the gradient alignment (correlation values) for each window and term. For clarity, we only present the relevant gradient correlations for each term as indicated by our prior cross-sectional analyses, though Figure S8 provides both. Age-windowing revealed relatively strong Gradient 3 correlations for baseline age across age windows above ~30 years of age, suggesting that a 25-year interval is long enough to capture this effect across most of the adult lifespan). Baseline pathology effects remained consistently aligned with Gradient 1 across all age windows beyond 40 years of age. The within-subject pathology change (ΔPathology) showed the strongest Gradient 1 alignment at approximately the same age window as baseline pathology, but decreased somewhat in older age.

Across the pathology windows, baseline age showed a decreasing association with Gradient 3, with weaker correlations in windows containing higher baseline pathology. Baseline pathology effects, in contrast, initially increased in alignment with Gradient 1 before declining at higher pathology levels, echoing findings from the cross-sectional GAM analysis. Interestingly, ΔPathology followed a similar trajectory but preceded the baseline pathology effect, peaking and declining in earlier pathology windows. While we observed an increase at the highest end of the baseline pathology windows, we also note limited sample size and reduced reliability of estimates at distributional extremes. Overall, these findings corroborate and extend our cross-sectional results, demonstrating that AD pathology-related FC changes have a temporal dynamic. Within-subject increases in tau pathology are initially associated with connectivity changes along Gradient 1, but this relationship weakens as baseline pathology advances. Moreover, the stronger ΔPathology effects at lower pathology windows, compared to baseline pathology, suggest that gradient-aligned connectivity changes may indicate emerging pathology.

### Gradient-aligned FC patterns reflect cognitive status

In the previous analyses, we linked gradient-like connectivity alterations to two factors known to affect cognition: age and AD. However, these analyses did not directly examine the relationship between connectivity and cognitive performance, which is essential for understanding the implications of these findings. Hence, to assess the relationship between cognition and FC alterations in our presented framework we examined the association between FC and cognitive performance in two groups: cognitively healthy, CSF Aβ42/40-negative, APOE ε4 non-carriers (N = 310) and individuals with mild cognitive impairment (MCI) or AD dementia (N = 258). This split was intended to assess whether the FC patterns are related to cognition in the absence of cognitive decline attributable to AD dementia. Cognitive performance was measured using a modified Preclinical Alzheimer Cognitive Composite (mPACC) score^47^, zero-normalized to a healthy control group over 60 (see Methods for details).

**Figure 5:**
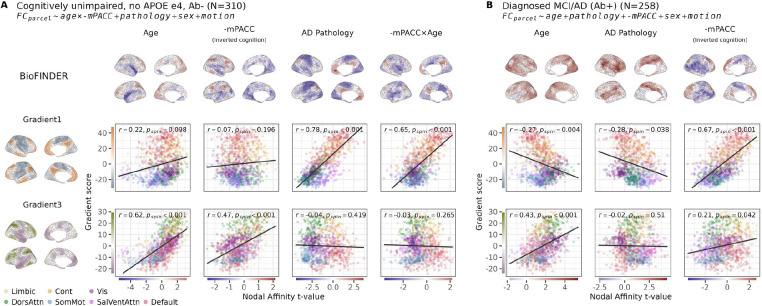
Cognitive status modifies the relationship between AD pathology, age, and functional connectivity changes along Gradient 1. Cross-sectional results are shown for (A) cognitively unimpaired Aβ−, APOE ε4 non-carriers and (B) cognitively impaired individuals with mild cognitive impairment (MCI) or AD dementia. In A, models include age, pathology, cognition (mPACC), and an cognition × age interaction (with centered predictors and inverted cognition scores for interpretability). In B, models include age, pathology, and cognition without interactions. Tau-driven AD pathology in the cognitively unimpaired group is associated with Gradient-1-like FC changes whereas it is absent in the MCI/AD dementia group. Instead, worse cognition (-mPACC in B) in the MCI/AD dementia group is associated with Gradient-1-like changes, which closely resembles the effects seen in older cognitively unimpaired individuals (−mPACC × Age in A).

In the healthy group, node-wise linear regression was conducted with mean centered age, (inverted) mPACC and their interaction as predictors. Due to common age-related tau accumulation^48^, we adjusted for pathology load in the healthy group (see Figure S9 for analyses without this adjustment and distribution of Tau SUVR within this group). Results (Figure 5 A) confirmed alignment of age effects with Gradient 3 (r = 0.62, p_spin_<0.001). Gradient 3-like changes in FC were also associated with worse cognition (r = 0.47, p_spin_<0.001). This means that, at the sample mean age (62 years), people with poorer cognition generally have decreases of FC in executive regions and less decreases or increasing FC in non-executive regions (i.e. have more of a Gradient 3-like FC pattern). This main effect was also present in the absence of the interaction term (Figure S10). The interaction between age and cognition, however, showed a Gradient 1-like pattern (r = 0.65, p_spin_<0.001) – meaning that older people with poor cognition generally have decreased FC in sensory-motor regions and less decreased or increased FC in associative regions (i.e., have more of a Gradient 1-like FC pattern). Surprisingly, we found that the AD pathology (in this case, early stage tau pathology, since all subjects in this group are are Aβ−) in the cognitively healthy group also aligned with Gradient 1 (r = 0.78, p_spin_<0.001), again suggesting that gradient-aligned connectivity changes may indicate emerging pathology.

In the cognitively impaired group, parcel-wise regression was performed to examine FC associations with AD pathology and (inverted) mPACC, adjusted for age. Surprisingly, the spatial distribution of AD effects no longer resembled a Gradient 1-like pattern in this group. Instead, a Gradient 1-like pattern emerged for worse cognition, regardless of pathology load or age (Figure 5 B, see also Figure S11 for the same subgroup analysis but without cognition). This suggests that in the clinical phase of AD, cognitive difficulties are more directly related to Gradient 1-aligned connectivity changes than those connectivity changes are to underlying pathology. Supplementary analyses (Figure S12) confirmed that adding an interaction between pathology and cognition did not alter this main effect.

## Discussion

This study demonstrates that age- and AD-related changes in functional connectivity are fundamentally shaped by macro-scale brain organization. Our findings offer a more holistic perspective of connectivity alterations free from constraints imposed by predefined region or network borders, allowing reinterpretation of prior, often contradictory, findings. We highlight that hyper- and hypoconnectivity rarely occur in isolation; instead, they coexist systematically, forming consistent spatial patterns that align with two distinct principal axes describing functional brain organization. More (global) AD pathology is associated with decreased FC in sensorimotor areas, and increased (or less decreased) FC in associative areas – aligning with the sensorimotor-association axes (also referred to as Gradient 1). Higher age is associated with decreased FC in non-executive areas, and increased (or less decreased) FC in executive areas – aligning with the executive axes (also referred to as Gradient 3). These results were replicated in an external cohort, confirmed longitudinally, and across multiple methodological choices. Further, we demonstrate that these organization-aligned spatial patterns shift with age and with severity of AD pathology. For instance, connectivity changes aligned with Gradient 1 emerge as an early response to AD pathology, even before clinically detectable impairment, but diminish once clinical impairment develops. At this later stage, Gradient 1 alignment more directly reflects worse cognitive performance rather than having higher AD pathology, possibly signaling cognitive aspects of disease progression. A similar relationship between Gradient 1-aligned connectivity and cognitive performance appeared in clinically unimpaired older adults. Taken together, our results suggest that connectivity changes aligned with organizational axes (specifically the sensorimotor-association axes) may serve as a neural indicator of cognitive strain even before clinical impairment becomes evident.

The cortical patterns of connectivity changes identified in this study illustrate a balancing scale of age-related connectivity increases and decreases, anchored at the centers of Gradients 1 and 3 for AD and aging, respectively. Connectivity changes in one functional domain, as captured by these gradients (e.g. decreased FC in sensorimotor areas for Gradient 1), are mirrored by less or opposing changes in its “opposite” domain (e.g. increased FC in associative areas for Gradient 1). This dynamic is particularly noteworthy when considering the cognitive domains affected by AD and aging, which align closely with Gradients 1 and 3, respectively. AD primarily impairs episodic memory, a function linked to the default mode and limbic networks^49^. These networks, together with the control network, are located on the end of Gradient 1 that, in this study, generally tended to show increased FC with early AD pathology. In contrast, aging in the absence of pathology leaves autobiographical memory relatively less affected^50^ and predominantly affects executive functions^51^, which are closely linked to the dorsal attention, control and salience networks^52^. These cognitive differences also mirror structural vulnerabilities, with AD primarily affecting medial temporal regions and normal aging primarily frontal regions^50,53^. The dorsal attention, control, and visual network are located at the end of Gradient 3 that, in this study, generally tended to show increased FC with age. This systematic reconfiguration of connectivity may be indicative of shifts in resource allocation^2^ and offers a cognitively meaningful interpretation of the gradients, extending their relevance beyond mere organization, to encompass functional or cognitive vulnerabilities or adaptations in the context of aging and AD.

The results from literature describing FC alterations in aging and AD are highly heterogeneous^30,46,54^. Our study suggests that previously observed region- or network-specific effects are likely manifestations of broader, structured changes. The structured, gradient-like changes we demonstrate overlap with consistent findings in the literature. For instance, hypoconnectivity within the default mode^10,55^ and salience networks – particularly in the opercular insula^30,56,57^ – has been frequently reported in healthy aging. Yet, hypoconnectivity within the dorsal attention network has also been reported^30^, which contrasts with the hyperconnectivity observed in the present study. This discrepancy might reflect the omission of age-related tau pathology as a covariate in typical healthy aging studies (although, our results remain largely unchanged when not controlling for age-related tau pathology: Figure S9). Indeed, we show here that tau levels affect FC even in cognitively normal, Aβ negative individuals, and are particularly associated with decreased connectivity in the dorsal attention network.

Regarding AD, we observed widespread pathology-related connectivity reductions in unimodal areas – a finding documented previously^58–60^, yet often overlooked, potentially due to a literature bias towards DMN-related changes^46^. Consistent with past studies^1,16,19,28,34,61^, we observed DMN hyperconnectivity in early and preclinical AD, which have been reported even in genetically predisposed individuals^62–65^. Conversely, DMN hypoconnectivity is also frequently reported^28,66–72^. Previous reports^6,28,34^ have suggested that initial hyperconnectivity early on in the disease may give way to hypoconnectivity as the disease advances, perhaps resolving this discrepancy. Although hyperconnectivity may initially serve an adaptive purpose, chronic hyperconnectivity has been argued to become detrimental due to sustained metabolic stress^2^ or facilitating the spread of pathology^6^. Indeed,^6^ suggest the hyperconnectivity to ultimately drive the hypoconnectivity observed in AD. Supporting this “phasic” view, our nonlinear analysis showed that gradient alignment peaked at moderate pathology levels before declining as pathology burden increased further. Our longitudinal analysis further reinforced this, demonstrating that within-subject pathology accumulation (ΔPathology) also induces gradient-aligned FC changes, but that this relationship disappears for individuals with higher baseline pathology.

Strikingly, the relationship between pathology load and Gradient 1-aligned connectivity was entirely absent in MCI and AD patients. Instead, in this group, having a lower cognitive performance was associated with a Gradient 1-like FC pattern. A similar relationship to cognition also emerged among clinically unimpaired older adults. Previous studies have linked hyperconnectivity in the DMN with subjective cognitive decline^73^, as well as sensory-motor hyperconnectivity with better cognition and hypoconnectivity with worse cognitive performance^74–76^. Together, these findings could suggest that the Gradient 1-related FC patterns we observed could indeed represent a harmful state, indicating increased vulnerability to pathological processes leading to cognitive decline. But it could alternatively be interpreted as individuals experiencing underlying cognitive difficulties – potentially due to comorbidities or less cognitive resilience – requiring greater compensatory adaptations. These interpretations are not mutually exclusive. Intervention studies with repeated imaging and detailed cognitive testing over time^18,20,77^ will be necessary to disentangle the temporal dynamics of these interactions to assess if the gradient-like FC alterations are compensatory, harmful, or both.

Regardless of interpretation, gradient-like FC alterations appear to signal ongoing physiological or pathological processes, potentially reflecting underlying cognitive strain. Although resting-state fMRI and FC have often been proposed as a biomarker for AD (e.g.^68^), its practical effectiveness remains limited^78^. Our findings suggest that examining whole-cortex patterns of FC, particularly using gradient alignment, may enhance the utility of fMRI as a biomarker. Gradient alignment does not seem to be disease-specific, but is associated with several factors that are directly or indirectly associated with cognition. If one could replicate the results on subject level, gradient alignment of FC could possibly work as a tool to detect and monitor clinically subtle cognitive changes. As indicated by the marked Gradient 1-alignment of AD pathology (driven by tau) in the cognitively unimpaired group, using gradient alignment as a biomarker might enable identification of early cognitive vulnerability before clinical symptoms emerge. Beyond this clinical implication, our results also offer conceptual clarity for FC research more broadly. Rather than viewing gradients as outcomes, this study demonstrates their value as interpretative frameworks for understanding how brain function adapts or responds to aging and disease processes.

The strength of this study lies in its large, single-site main cohort and the several methods employed to validate the findings within it, including an external replication, nonlinear, and longitudinal linear and nonlinear analyses. The longitudinal approach of this paper should be emphasized, as such within-subject designs still are relatively rare in the field^78^. By using a whole-brain perspective and continuous measures of AD pathology, we also overcome the limitations of region-specific analyses and categorical diagnostic classifications. Using several different methodological approaches we show that the results are generally not methodology dependent, but subtle differences between e.g. nodal affinity and nodal connectivity strength may be worth further investigation. However, this study also has several limitations. First, combining multiple pathology measures to map a disease trajectory obscures potential pathology-specific effects that could have been of interest. Second, our whole-brain approach overcomes biases inherent in region- or network-specific analyses but limits the ability to pinpoint specific connections driving the gradient-aligned patterns. Third, resting-state connectivity has a weaker relationship to cognition compared to task-based paradigms^75,79^; future studies should explore whether task-based FC strengthens or refines the findings of this study. Lastly, as an observational study, causal inferences regarding the relationship between functional connectivity, pathology, and cognitive decline is limited.

In sum, our findings reveal that age- and AD-related FC alterations align with distinct large-scale cortical gradients of functional organization. These alignments vary along the age and AD pathology spectra, and are associated with cognition, independent of AD pathology burden. We propose that gradient-like FC changes may reflect a general neural response to cognitive strain or system-level stress. However, whether this response is adaptive or compensatory remains unknown. We show that these patterns emerge in older individuals, those with subtle AD pathology, and clinically impaired patients alike, suggesting that cognitive demands may increasingly exceed available neural resources in these groups.

## Methods

### Sample

#### BioFINDER.

Information regarding recruitment, diagnostic criteria, and Aβ positivity assessment for the BioFINDER-2 cohort (https://biofinder.se/two; clinical trial number NCT03174938) has been described in detail previously^80,81^. All participants provided written informed consent in accordance with the Declaration of Helsinki. Ethical approval for the study was obtained from the Ethics Committee of Lund University, Lund, Sweden, with additional permissions for PET imaging granted by the Swedish Medicines and Products Agency and the local Radiation Safety Committee at Skåne University Hospital, Sweden. For the cross-sectional analysis, 1,168 individuals had all relevant data available (fMRI, CSF Aβ42/40, and tau PET). However, as described in the Imaging section, a stringent in-scanner motion filter was applied, excluding 194 individuals and leaving a final cross-sectional sample of N = 973. Of these, 655 were cognitively unimpaired or had self-reported cognitive decline (SCD), while 319 were diagnosed with mild cognitive impairment or dementia^82^. Cognitively impaired cases were included only if they were Aβ-positive, as assessed by abnormal CSF Aβ42/40 values. For the longitudinal analysis, 480 individuals had fMRI and tau PET data from at least two visits. After applying the motion filter, 378 unique individuals with at least two data points remained. See tables Table 1 and Table 2 for additional details.

#### ADNI.

Information related to participant consent in The Alzheimer’s Disease Neuroimaging Initiative can be found at (ADNI; http://adni.loni.usc.edu). For up-to-date information, see www.adni-info.org. A total sample of N = 131 had the exact same variables available as in the discovery cohort (CSF Aβ42/40, tau PET, fMRI), with two individuals being excluded for excessive in-scanner motion. Of those, there were 89 healthy controls and 40 participants diagnosed with dementia or mild cognitive impairment. All cognitively impaired cases were Aβ-positive as assessed by abnormal CSF Aβ42/40 or amyloid PET. See table Table 1 for further details.

### Data acquisition and processing

#### CSF and APOE ε4 carriership

##### BioFINDER:

CSF Aβ42 and Aβ40 levels in BioFINDER were measured using the Roche Elecsys platform (Roche Diagnostics International Ltd., Basel, Switzerland). Aβ positivity was determined by Gaussian mixture modeling, using a cutoff of <0.080, previously described in^81,83^. Participants who carried at least one copy of the APOE ε4 allele were defined as APOE ε4 carriers.

##### ADNI:

CSF Aβ42 and Aβ40 levels in ADNI were measured using the Roche Elecsys platform with Elecsys CSF immunoassays on a cobase 601 analyzer at the University of Pennsylvania, as described in^84^. Aβ positivity was determined using previously established amyloid-PET cut-offs derived in^85^. APOE genotyping was performed using blood samples, and participants carrying at least one APOE ε4 allele were classified as APOE ε4 carriers. See^84^ for further details on CSF analysis and APOE genotyping in ADNI.

#### Cognitive assessment

Estimation of cognitive performance using the modified Preclinical Alzheimer’s Cognitive Composite (mPACC^47^) in BioFINDER has been previously described^82^. It is the average of five z-scores from the cognitive tests: Alzheimer Disease Assessment Scale (ADAS) delayed recall (weighted double), Animal Fluency, Mini-Mental State Examination (MMSE) and Symbol Digit Modalities Test (SDMT).

#### Pathology score

As with our previous work^86^, a pathology score representing accumulated AD neuropathology was calculated using the R package SCORPIUS^45^. The variables included were the CSF Aβ42/40-ratio, together with PET SUVR from regions reflecting Braak I-II, III-IV and V-IV as defined by^87^. SCORPIUS is a trajectory inference algorithm designed to nonlinearly project high-dimensional data onto a single continuous path. Briefly, pairwise Euclidean distances between observations were calculated. The resultant distance matrix was then decomposed and three components retained. Each individual is hence represented by coordinates in this three-dimensional space. Observations were then clustered into four clusters using k-means and the shortest path going through all cluster centers found. A principal curve was then iteratively fitted to the data to model the progression trajectory. Methodological parameters, such as the number of components and cluster centers, were set to the default values used in SCORPIUS. Each participant was projected onto this curve to assign a pathology score reflecting its position along the inferred trajectory. See Figure 1 D for estimation of the pathology measures as a function of the combined pathology score and distribution of patient groups over it. For the longitudinal pathology score, since longitudinal CSF Aβ40/42 data was largely unavailable, only tau PET SUVR from the three Braak meta ROIs (I-II, III-IV and V-VI) were used.

### Imaging

#### MRI and Tau PET

##### BioFINDER.

Structural MRI was acquired using a Siemens 3T MAGNETOM Prisma scanner (Siemens Healthineers, Erlangen, Germany) with a 64-channel head coil. T1-weighted images (Magnetization Prepared – Rapid Gradient Echo, MPRAGE) were acquired with the following parameters: in-plane resolution = 1 × 1 mm^2^, slice thickness = 1 mm, repetition time (TR) = 1900 ms, echo time (TE) = 2.54 ms, flip-angle = 9°.

All resting-state fMRI data (eyes closed) were acquired using a 3D echo-planar imaging (EPI) sequence with an in-plane resolution of 3×3 mm and slice thickness 3.6 mm; echo time = 30ms, and flip-angle = 63°. Scan time was 7.85 minutes, with a multiband repetition time of 1020 ms, resulting in 462 frames per scan before processing and censoring. Preprocessing has briefly been described in^26^. The processing was performed using a modified CPAC^88^ pipeline, building mostly on FSL^89^, AFNI^90^ and ANTS^91^. Skullstripping was done with in-house code using the T2 structural image as a primer and Brain Surface Extractor from BrainSuite^92^. The fMRI preprocessing included slice-timing and motion correction. Physiological noise was regressed out using CompCor^93^, alongside the removal of linear and quadratic trends. Additionally, regression of Friston’s 24-parameter motion correction^94^, white matter and CSF signals were performed. Susceptibility distortion was corrected by unwarping the functional data using a nonlinear diffeomorphic transformation (performed with ANTS) of the mean functional image to high resolution (1 × 1 × 1 mm3) T2 structural image^95^. This transformation was then applied to each volume in the fMRI timeseries. Finally, a bandpass filter (0.01–0.1 Hz) was applied, and images were transformed from native to MNI space with ANTS. For each scan, frames were censored based on DVARS for that frame lying outside 1.5*IQR above and below the third and first quartiles, respectively^96^. To avoid distortion from the outlier frames, they were interpolated before bandpass filtering and then removed from the final 4D image. Finally, any participant with an average FD > 0.3mm or maximum FD > 3mm over the entire sequence were filtered out before analysis.

Tau PET acquisition has been described in detail by^97^. Briefly, participants were injected with an average of 365 ± 20 MBq [18F]RO948, and emission data was acquired between 70 and 90 minutes post-injection, adjusted for the tracer’s pharmacokinetics. Low-dose CT scans were conducted before PET scans for attenuation correction. PET data was reconstructed using the VPFX-S algorithm (ordered subset expectation maximization with time-of-flight and point spread function corrections). The LIST mode data was binned into 4 × 5-minute time frames, and images were motion-corrected, summed, and co-registered to corresponding T1-weighted MRI images. FreeSurfer parcellation (v6; http://surfer.nmr.mgh.harvard.edu.ludwig.lub.lu.se/) was applied to extract regional uptake values, normalized to the mean uptake in inferior cerebellar grey matter.

##### ADNI.

Image processing for ADNI has been described in^98^. Briefly, MRI data were collected using 3T scanners with unified scanning protocols that can be accessed here: https://adni.loni.usc.edu/wp-content/uploads/2017/07/ADNI3-MRI-protocols.pdf. Structural MRI was acquired with a 3D T1-weighted MPRAGE sequence (1 mm isotropic voxels, TR = 2300 ms).

Two protocols exist for the resting state EPI-BOLD sequence in ADNI: Basic and Advanced. Depending on which protocol a participant has been subjected to, the parameters vary. Both protocols have an approximate scanner time of 10 minutes. The Basic protocol has a TR/TE/flip angle=3000/30/90° and the Advanced a TR/TE/flip angle=600/30/53°, so the number of frames differ depending on the protocol and ranges from approximately 200 to 1000. Processing steps described in^98^ included motion correction, as well as regression of six motion parameters and mean signal from CSF and white matter. Trends were removed and a (0.01–0.08Hz) band-pass filter was applied. Images were nonlinearly registered to MNI space with coregistration to the baseline T1 image. After timeseries had been extracted, rows from high motion frames (> 0.5mm framewise displacement) were censored. These rows, together with one preceding and two subsequent, were removed. Out of the 131 subjects with a full set of variables (CSF Aβ 40/42, tau PET, fMRI), one individual was filtered out due to a maximum number of frames < 100 and one participant with an average FD > 0.3mm or maximum FD > 3mm over the entire sequence was filtered out before analysis.

Tau PET imaging in ADNI3 was performed using [^18F]AV-1451 (Flortaucipir). Participants received an injection of 370 MBq (±10%), and emission data was acquired 75 minutes post-injection. Scans were conducted for 30 minutes in six 5-minute frames. Images were reconstructed using iterative algorithms specific to each scanner model, incorporating corrections for scatter, attenuation, and motion artifacts. CT scans or transmission scans were performed for attenuation correction prior to emission imaging. More detailed information can be accessed here https://adni.loni.usc.edu/wp-content/uploads/2012/10/ADNI3_PET-Tech-Manual_V2.0_20161206.pdf.

#### Assessment of functional connectivity

In both BioFINDER and ADNI, parcel-wise timeseries were extracted using the 1000-region Schaefer atlas^43^, a cortical parcellation based on functional connectivity patterns and spatial contiguity. The timeseries were smoothed using a full-width at half-maximum kernel of 6 mm in BioFINDER and 4 mm in ADNI. After mean centering and scaling the raw timeseries, pairwise Pearson correlations were computed between parcels, resulting in 1000×1000 correlation matrices representing co-activation patterns. Negative correlations were set to zero. FC was quantified primarily as nodal affinity^35^, where each connectome was column-wise thresholded to retain the 25% strongest connections, and cosine similarity was calculated between columns to yield a matrix of connectivity similarity. This approach shifts the interpretation slightly from similarity of brain activity to similarity of connectivity patterns, however, these two are very closely aligned in practice.^35^ used a threshold retaining the 10% strongest connections, while we opted for a more liberal threshold due to the lower spatial resolution in our clinical data and coarser brain parcellation of using 1000 parcels instead of surface vertices.

Nodal affinity reduces the rank of the original correlation matrix, highlighting dominant patterns of variance while preserving interpretability. Averaging the affinity matrix across parcels then yields a metric that reflects how generic or specialized a parcel’s connectivity profile is relative to the rest of the brain: higher values indicate widespread similarity, whereas lower values point to more distinct, unique connectivity patterns. Affinity is widely used in functional gradient studies because it is traditionally the underlying matrix from which the gradients are derived^35^. For comparison, nodal connectivity strength – the parcel-wise average of the original connectivity matrices – was also calculated. All main analyses were replicated with nodal strength as the outcome, yielding results comparable to those with nodal affinity (see Figure S1). Additionally, we replicated our findings using correlation instead of cosine similarity on unthresholded connectivity matrices, which also yielded comparable results (see Figure S13). In Figure S6 and Figure S7, we run the main analyses with nodal affinity calculated within and between each Yeo 7 network, respectively, by taking the average affinity values of each parcel within its own network, and the average of each parcel’s affinity to parcels outside of its own network.

#### Derivation of functional gradients

Breaking down the connectivity matrices into low-dimensional components, known as gradients^35^, captures connectivity patterns of the brain as continuous measures of connectivity similarity over the cortex. Regions with similar connectivity patterns will get grouped together at different ends of these gradients. The primary components in such analyses represent the major variance axes of functional connectivity. For BioFINDER and ADNI, respectively, we calculated an estimate of the functional connectome by averaging individual connectivity matrices from cognitively unimpaired APOE ε4 non-carriers without abnormal Aβ levels. In BioFINDER, only younger participants (≤60 years) were included (n = 125), whereas in ADNI, the entire age range was used (n = 82) due to the unavailability of a younger sample.

The variance components, or gradients, of these two connectomes were derived using principal component analysis (PCA) with singular value decomposition directly on the connectome without thresholding. Traditionally, gradients are derived using diffusion map embedding^35,99^, which involves applying heavy thresholding to the connectome before taking the pairwise cosine similarity of the rows/columns. However, we found that both methods produced comparable components with respect to the original gradients from^35^ (accessed from https://identifiers.org/neurovault.collection:1598). We felt that applying PCA directly on the connectome, without transformation or thresholding, offered a simpler and more interpretable approach compared to diffusion map embedding, and did not result in a notable divergence. In Figure S2, we compare the two methods over a set of thresholding values, supporting this decision (see also^100^ for a more formal comparison). Supplementary sensitivity analyses using gradients derived from both diffusion map embedding and PCA over different thresholds confirmed that this choice did not alter our results to any great extent, see Table S1.

### Statistical analyses

For the cross-sectional analyses, linear regression models were run for each parcel, with the functional connectivity metric as outcome, and the terms of interest as predictors, covaried for sex and motion (framewise displacement). For example, for the analyses looking at age and AD pathology, the model formula was as follows: *FC*_*parcel*_ ~ *age* + *pathology* + *sex* + *motion*. This was repeated for each of the 1000 parcels. The t-maps were then compared to the gradients using Pearson correlation. To assess the spatial correspondence between these two maps, while accounting for spatial autocorrelation, we performed a spin test based on the method introduced by^44^. Using the Hungarian algorithm, we generated 1000 random rotations of the parcel centroid coordinates, separately rotating the left and right hemispheres while preserving their anatomical structure. For each rotation, parcels were reassigned by minimizing the Euclidean distance between original and rotated centroids. We then recomputed the correlation between the rotated maps and compared the empirical correlation against the null distribution to obtain a permutation-based p-value.

To investigate cross-sectional nonlinearities in the relationship between functional connectivity and age or pathology, generalized additive models (GAMs) with penalized regression splines (thin plate) were fitted for each parcel, with age and pathology as smooth terms and sex and motion as linear covariates (*FC*_*parcel*_ ~ *s* (*age*) + *s* (*pathology*) + *sex* + *motion*). The models were fitted using the R package mgcv (version 1.9–1, Wood 2011a), with default values for the number k basis functions. The first derivatives of the smooth terms were calculated at 100 evenly distributed points along each spectrum using finite differences, capturing the slope and effect direction at specific ages and pathology loads. For age, these derivatives were calculated over the central 90% of the range. These derivatives were then used to calculate R^2^ values by correlating the slopes at each point with Gradient 1 (for pathology) or Gradient 3 (for age) scores. Gradient alignment could then be assessed continuously across the age and pathology spectra. To visualize these effects, derivatives were averaged over quartiles of pathology and age. The resulting average slopes were mapped onto the cortical surface. Additionally, FC metric predictions were generated across the same ranges, holding age constant at the sample mean (66.5) when assessing pathology effects and pathology constant at 0.1 when assessing age effects. Parcels were grouped into 20 equally sized bins (ventiles) based on Gradient 1 (for pathology) or Gradient 3 (for age) scores, and predicted values within each ventile were averaged.

For the longitudinal analysis, linear mixed-effects models (lme4^101^) were fit at each parcel with FC as the outcome and baseline age, baseline pathology, and within-subject pathology change (Δ pathology) as fixed effects. Time between baseline and follow-up scan, sex and scanner motion were included as (fixed effects) covariates of no interest, and random intercepts were specified for subjects, i.e. *FC*_*parcel*_ ~ *age*_*baseline*_ + *pathology*_*baseline*_ + *Δpathology*_*ti–t0*_ + *time* + *sex* + *motion* + (*1*|*subject*). The within subject change in pathology was calculated as the difference between the baseline pathology score and the pathology score at each follow up time point for each individual. This approach separates the between and within subject effects. Obtained parcelwise t-values from each fixed effect of interest were then correlated with the parcelwise component scores of the gradients.

To assess nonlinear patterns of longitudinal change across the old age and AD pathology spectrum, we employed a sliding window approach. This allowed us to detect localized changes without smoothing over transient effects. In this approach the model described in the previous paragraph was fitted at the parcel level repeatedly in windows traversing over baseline age and baseline pathology separately (i.e. age windows were only filtered on age and vice versa). Age windows were set at 25 years and incremented by 1 year, while pathology windows spanned 0.35 pathology score units and were incremented by 0.015, resulting in 44 and 45 windows respectively. Although relatively arbitrary, the window size of age was chosen to balance the number of subjects in each window, being sufficiently wide to capture the age effects we observed across the whole sample while being sufficiently narrow to capture nonlinearities across it. This window size turned out to be approximately 35% of the full age range, hence the pathology window was set at 35% of the pathology range [0, 1] as well. The pathology increment was chosen to get approximately the same number of windows as for age. Obtained t-values for each fixed effect of interest, in each window, were correlated with gradient scores. This allowed for a dynamic assessment of gradient alignment across both the baseline age and pathology spectra.

## Supplementary Material

Supplement 1

## Figures and Tables

**Table 1:** Cross-sectional characteristics in BioFINDER and ADNI.

Cohort			CN	MCI Aβ+	AD

BioFINDER	Total N (%)		655 (67.3)	152 (15.6)	166 (17.1)
	Age	Mean (SD)	63.43 (14.59)	72.55 (7.44)	72.50 (8.10)
	Pathology Score Score	Mean (SD)	0.14 (0.13)	0.41 (0.23)	0.67 (0.22)
	Aβ	Negative	474 (72.4)		
		Positive	181 (27.6)	152 (100.0)	166 (100.0)
	Braak12 (SUVR)	Mean (SD)	1.18 (0.23)	1.65 (0.42)	1.99 (0.41)
	Braak34 (SUVR)	Mean (SD)	1.17 (0.17)	1.55 (0.46)	2.17 (0.68)
	Braak56 (SUVR)	Mean (SD)	1.05 (0.09)	1.20 (0.27)	1.55 (0.44)
	Mean FD (mm)	Mean (SD)	0.16 (0.06)	0.18 (0.06)	0.18 (0.05)
	Education (Years)	Mean (SD)	13.06 (3.37)	13.19 (4.41)	12.47 (3.98)
	Sex	Female	357 (54.5)	65 (42.8)	88 (53.0)
		Male	298 (45.5)	87 (57.2)	78 (47.0)
ADNI	Total N (%)		89 (69.0)	18 (14.0)	22 (17.1)
	Age	Mean (SD)	74.25 (6.44)	74.37 (7.14)	77.41 (7.47)
	Pathology Score Score	Mean (SD)	0.09 (0.11)	0.26 (0.19)	0.47 (0.24)
	Aβ	Negative	51 (57.3)		
		Positive	38 (42.7)	18 (100.0)	22 (100.0)
	Braak12 (SUVR)	Mean (SD)	1.09 (0.13)	1.33 (0.24)	1.53 (0.31)
	Braak34 (SUVR)	Mean (SD)	1.10 (0.09)	1.23 (0.18)	1.47 (0.32)
	Braak56 (SUVR)	Mean (SD)	1.07 (0.08)	1.16 (0.16)	1.41 (0.39)
	Mean FD (mm)	Mean (SD)	0.10 (0.05)	0.08 (0.04)	0.11 (0.06)
	Education (Years)	Mean (SD)	16.63 (2.36)	16.22 (2.34)	15.95 (2.59)
	Sex	Female	53 (59.6)	7 (38.9)	11 (50.0)
		Male	36 (40.4)	11 (61.1)	11 (50.0)

Pathology scores were calculated by non-linearly mapping individuals’s CSF Aβ42/40 ratio and tau PET SUVR from Braak I-II, III-IV, V-VI regions onto a trajectory from 0 to 1 where 0 indicates no pathology.

Abbreviations: FD = frame displacement (scanner motion); SD = standard deviation, CN = cognitively normal; MCI = mild cognitive impairment; AD = Alzheimer’s disease.

**Table 2:** Characteristics of the longitudinal BioFINDER-2 sample.

TimePoint			CN	MCI Aβ+	AD

Constant	Total N (%)		287 (75.9)	54 (14.3)	37 (9.8)
	Number of visits	2	231 (80.5)	51 (94.4)	37 (100.0)
		3	55 (19.2)	3 (5.6)	0 (0.0)
		4	1 (0.3)	0 (0.0)	0 (0.0)
	Follow up (Years)	Mean (SD)	2.49 (0.93)	2.17 (0.78)	1.75 (0.25)
	ΔPathology score	Mean (SD)	0.02 (0.07)	0.06 (0.08)	0.06 (0.05)
	Sex	Female	151 (52.6)	23 (42.6)	17 (45.9)
		Male	136 (47.4)	31 (57.4)	20 (54.1)
	Education (Years)	Mean (SD)	13.09 (3.58)	12.29 (4.61)	12.21 (3.70)
Baseline	Age	Mean (SD)	61.56 (14.41)	72.44 (7.95)	72.35 (7.85)
	Pathology score	Mean (SD)	0.15 (0.12)	0.42 (0.22)	0.64 (0.19)
	aβ	Negative	212 (74.6)		
		Positive	72 (25.4)	53 (100.0)	37 (100.0)
	Braak12 (SUVR)	Mean (SD)	1.16 (0.22)	1.61 (0.39)	1.98 (0.40)
	Braak34 (SUVR)	Mean (SD)	1.16 (0.17)	1.55 (0.48)	2.03 (0.66)
	Braak56 (SUVR)	Mean (SD)	1.05 (0.10)	1.21 (0.36)	1.47 (0.45)
Last follow up	Age	Mean (SD)	64.22 (14.50)	74.91 (7.89)	74.48 (7.85)
	Pathology score	Mean (SD)	0.17 (0.14)	0.48 (0.23)	0.70 (0.19)
	Braak12 (SUVR)	Mean (SD)	1.20 (0.25)	1.73 (0.41)	2.03 (0.39)
	Braak34 (SUVR)	Mean (SD)	1.19 (0.23)	1.75 (0.65)	2.25 (0.73)
	Braak56 (SUVR)	Mean (SD)	1.06 (0.13)	1.33 (0.52)	1.62 (0.55)

Because no CSF Aβ data were available longitudinally for AD dementia patients, pathology scores were recomputed using only tau PET SUVR from Braak I-II, III-IV, V-VI regions. TimePoint indicates whether a variable is baseline, last available follow-up, or consistent across time.

Abbreviations: FD = frame displacement (scanner motion); SD = standard deviation, CN = cognitively normal; MCI = mild cognitive impairment; AD = Alzheimer’s disease.

## Data Availability

Data from the validation cohort ADNI is a public access dataset and can be obtained by application from http://adni.loni.usc.edu/. The BioFINDER data are not publicly available, but anonymized data may be made available upon request to qualified academic investigators. Data sharing must comply with EU General Data Protection Regulation (GDPR), decisions of the Swedish Ethical Review Authority, and regulations of Region Skåne.
