# Supplementary material for: Age and Alzheimer’s disease affect functional connectivity along separate axes of functional brain organization
Source: bioRxiv. 2025 May 25:2025.05.22.655469. Preprint. [Version 1] doi: 10.1101/2025.05.22.655469 (PMC12258722; doi:10.1101/2025.05.22.655469)
Supplement: Supplement 1 [file NIHPP2025.05.22.655469v1-supplement-1.pdf]

bioRxiv preprint doi: https://doi.org/10.1101/2025.05.22.655469; this version posted May 25, 2025. The copyright holder for this preprint (which was not certified by peer review) is the author/funder, who has granted bioRxiv a license to display the preprint in perpetuity. It is made available under aCC-BY 4.0 International license.

Supplementary

Model	Cohort	Filter	Method	Thresh	Term	Gradient 1	Gradient 3
FC ~ age + pathology	BioFINDER	Full sample	PCA	0.00	Age	$r = 0.22$, $p_{spin} = 0.080$	$r = 0.75$, $p_{spin} < 0.001$
					Pathology	$r = 0.74$, $p_{spin} < 0.001$	$r = 0.24$, $p_{spin} = 0.014$
				0.25	Age	$r = 0.2$, $p_{spin} = 0.083$	$r = 0.77$, $p_{spin} < 0.001$
					Pathology	$r = 0.76$, $p_{spin} < 0.001$	$r = 0.13$, $p_{spin} = 0.176$
				0.50	Age	$r = 0.23$, $p_{spin} = 0.071$	$r = 0.61$, $p_{spin} < 0.001$
					Pathology	$r = 0.75$, $p_{spin} < 0.001$	$r = 0.25$, $p_{spin} = 0.003$
				0.75	Age	$r = 0.46$, $p_{spin} = 0.004$	$r = 0.45$, $p_{spin} < 0.001$
					Pathology	$r = 0.54$, $p_{spin} < 0.001$	$r = 0.31$, $p_{spin} = 0.002$
			DME	0.00	Age	$r = 0.18$, $p_{spin} = 0.124$	$r = 0.8$, $p_{spin} < 0.001$
					Pathology	$r = 0.67$, $p_{spin} < 0.001$	$r = 0.25$, $p_{spin} = 0.016$
				0.25	Age	$r = 0.19$, $p_{spin} = 0.089$	$r = 0.8$, $p_{spin} < 0.001$
					Pathology	$r = 0.73$, $p_{spin} < 0.001$	$r = 0.2$, $p_{spin} = 0.072$
				0.50	Age	$r = 0.21$, $p_{spin} = 0.070$	$r = 0.69$, $p_{spin} < 0.001$
					Pathology	$r = 0.76$, $p_{spin} < 0.001$	$r = 0.37$, $p_{spin} < 0.001$
				0.75	Age	$r = 0.31$, $p_{spin} = 0.020$	$r = 0.52$, $p_{spin} < 0.001$
					Pathology	$r = 0.71$, $p_{spin} < 0.001$	$r = 0.38$, $p_{spin} < 0.001$
	ADNI		PCA	0.00	Age	$r = 0.09$, $p_{spin} = 0.336$	$r = 0.68$, $p_{spin} < 0.001$
					Pathology	$r = 0.54$, $p_{spin} < 0.001$	$r = 0.31$, $p_{spin} = 0.001$
				0.25	Age	$r = 0.09$, $p_{spin} = 0.342$	$r = 0.67$, $p_{spin} < 0.001$
					Pathology	$r = 0.54$, $p_{spin} < 0.001$	$r = 0.3$, $p_{spin} = 0.002$
				0.50	Age	$r = 0.1$, $p_{spin} = 0.315$	$r = 0.65$, $p_{spin} < 0.001$

bioRxiv preprint doi: https://doi.org/10.1101/2025.05.22.655469; this version posted May 25, 2025. The copyright holder for this preprint (which was not certified by peer review) is the author/funder, who has granted bioRxiv a license to display the preprint in perpetuity. It is made available under aCC-BY 4.0 International license.

					Pathology	r = 0.56, p_{spin} < 0.001	r = 0.28, p_{spin} = 0.002
				0.75	Age	r = 0.14, p_{spin} = 0.328	r = 0.68, p_{spin} < 0.001
					Pathology	r = 0.62, p_{spin} < 0.001	r = 0.29, p_{spin} < 0.001
			DME	0.00	Age	r = 0.14, p_{spin} = 0.146	r = 0.64, p_{spin} < 0.001
					Pathology	r = 0.52, p_{spin} < 0.001	r = 0.3, p_{spin} = 0.001
				0.25	Age	r = 0.13, p_{spin} = 0.154	r = 0.64, p_{spin} < 0.001
					Pathology	r = 0.53, p_{spin} < 0.001	r = 0.3, p_{spin} = 0.001
				0.50	Age	r = 0.14, p_{spin} = 0.181	r = 0.62, p_{spin} < 0.001
					Pathology	r = 0.54, p_{spin} < 0.001	r = 0.26, p_{spin} = 0.001
				0.75	Age	r = 0.16, p_{spin} = 0.256	r = 0.63, p_{spin} < 0.001
					Pathology	r = 0.59, p_{spin} < 0.001	r = 0.24, p_{spin} = 0.005
FC ~ age*(-)mPACC + pathology	BioFINDER	cognitively healthy	PCA	0.00	Age	r = 0.22, p_{spin} = 0.098	r = 0.62, p_{spin} < 0.001
					-mPACC	r = 0.07, p_{spin} = 0.196	r = 0.47, p_{spin} < 0.001
					Pathology	r = 0.78, p_{spin} < 0.001	r = -0.04, p_{spin} = 0.419
					Age × -mPACC	r = 0.65, p_{spin} < 0.001	r = -0.03, p_{spin} = 0.265
				0.25	Age	r = 0.21, p_{spin} = 0.094	r = 0.66, p_{spin} < 0.001
					-mPACC	r = 0.09, p_{spin} = 0.158	r = 0.37, p_{spin} < 0.001
					Pathology	r = 0.8, p_{spin} < 0.001	r = -0.11, p_{spin} = 0.249
					Age × -mPACC	r = 0.64, p_{spin} < 0.001	r = 0.03, p_{spin} = 0.543
				0.50	Age	r = 0.21, p_{spin} = 0.115	r = 0.5, p_{spin} < 0.001
					-mPACC	r = 0.1, p_{spin} = 0.130	r = 0.54, p_{spin} < 0.001
					Pathology	r = 0.78, p_{spin} < 0.001	r = -0.02, p_{spin} = 0.458
					Age × -mPACC	r = 0.61, p_{spin} < 0.001	r = -0.1, p_{spin} = 0.098
				0.75	Age	r = 0.41, p_{spin} = 0.034	r = 0.34, p_{spin} = 0.002
					-mPACC	r = 0.02, p_{spin} = 0.444	r = 0.6, p_{spin} < 0.001
					Pathology	r = 0.56, p_{spin} < 0.001	r = 0, p_{spin} = 0.516
					Age × -mPACC	r = 0.52, p_{spin} < 0.001	r = -0.11, p_{spin} = 0.088
			DME	0.00	Age	r = 0.2, p_{spin} = 0.114	r = 0.68, p_{spin} < 0.001
					-mPACC	r = -0.01, p_{spin} = 0.510	r = 0.38, p_{spin} < 0.001
					Pathology	r = 0.71, p_{spin} < 0.001	r = -0.05, p_{spin} = 0.396
					Age × -mPACC	r = 0.68, p_{spin} < 0.001	r = 0.04, p_{spin} = 0.474
				0.25	Age	r = 0.21, p_{spin} = 0.094	r = 0.69, p_{spin} < 0.001
					-mPACC	r = 0.03, p_{spin} = 0.314	r = 0.34, p_{spin} < 0.001
					Pathology	r = 0.76, p_{spin} < 0.001	r = -0.09, p_{spin} = 0.304

bioRxiv preprint doi: https://doi.org/10.1101/2025.05.22.655469; this version posted May 25, 2025. The copyright holder for this preprint (which was not certified by peer review) is the author/funder, who has granted bioRxiv a license to display the preprint in perpetuity. It is made available under aCC-BY 4.0 International license.

					Age × -mPACC	$r = 0.68, p_{spin} < 0.001$	$r = 0.09, p_{spin} = 0.252$
				0.50	Age	$r = 0.22, p_{spin} = 0.078$	$r = 0.58, p_{spin} < 0.001$
					-mPACC	$r = 0.07, p_{spin} = 0.207$	$r = 0.5, p_{spin} < 0.001$
					Pathology	$r = 0.77, p_{spin} < 0.001$	$r = 0.01, p_{spin} = 0.414$
					Age × -mPACC	$r = 0.68, p_{spin} < 0.001$	$r = 0, p_{spin} = 0.560$
				0.75	Age	$r = 0.3, p_{spin} = 0.041$	$r = 0.43, p_{spin} < 0.001$
					-mPACC	$r = 0.06, p_{spin} = 0.248$	$r = 0.58, p_{spin} < 0.001$
					Pathology	$r = 0.72, p_{spin} < 0.001$	$r = 0.02, p_{spin} = 0.416$
					Age × -mPACC	$r = 0.67, p_{spin} < 0.001$	$r = -0.04, p_{spin} = 0.318$
FC ~ age + pathology + -mPACC		impaired (MCI and AD)	PCA	0.00	Age	$r = -0.27, p_{spin} = 0.004$	$r = 0.43, p_{spin} < 0.001$
					Pathology	$r = -0.28, p_{spin} = 0.038$	$r = -0.02, p_{spin} = 0.510$
					-mPACC	$r = 0.67, p_{spin} < 0.001$	$r = 0.21, p_{spin} = 0.042$
				0.25	Age	$r = -0.3, p_{spin} = 0.002$	$r = 0.49, p_{spin} < 0.001$
					Pathology	$r = -0.25, p_{spin} = 0.064$	$r = -0.18, p_{spin} = 0.126$
					-mPACC	$r = 0.67, p_{spin} < 0.001$	$r = 0.23, p_{spin} = 0.064$
				0.50	Age	$r = -0.23, p_{spin} = 0.025$	$r = 0.3, p_{spin} = 0.002$
					Pathology	$r = -0.22, p_{spin} = 0.108$	$r = 0.11, p_{spin} = 0.088$
					-mPACC	$r = 0.66, p_{spin} < 0.001$	$r = 0.15, p_{spin} = 0.062$
				0.75	Age	$r = 0.07, p_{spin} = 0.383$	$r = 0.2, p_{spin} = 0.081$
					Pathology	$r = -0.4, p_{spin} = 0.002$	$r = 0.25, p_{spin} = 0.002$
					-mPACC	$r = 0.59, p_{spin} < 0.001$	$r = 0.08, p_{spin} = 0.136$
			DME	0.00	Age	$r = -0.25, p_{spin} = 0.008$	$r = 0.54, p_{spin} < 0.001$
					Pathology	$r = -0.33, p_{spin} = 0.003$	$r = -0.07, p_{spin} = 0.300$
					-mPACC	$r = 0.64, p_{spin} < 0.001$	$r = 0.24, p_{spin} = 0.031$
				0.25	Age	$r = -0.27, p_{spin} = 0.004$	$r = 0.53, p_{spin} < 0.001$
					Pathology	$r = -0.28, p_{spin} = 0.030$	$r = -0.13, p_{spin} = 0.195$
					-mPACC	$r = 0.66, p_{spin} < 0.001$	$r = 0.26, p_{spin} = 0.031$
				0.50	Age	$r = -0.27, p_{spin} = 0.004$	$r = 0.39, p_{spin} < 0.001$
					Pathology	$r = -0.25, p_{spin} = 0.061$	$r = 0.14, p_{spin} = 0.064$
					-mPACC	$r = 0.66, p_{spin} < 0.001$	$r = 0.22, p_{spin} = 0.009$
				0.75	Age	$r = -0.16, p_{spin} = 0.058$	$r = 0.25, p_{spin} = 0.028$
					Pathology	$r = -0.33, p_{spin} = 0.010$	$r = 0.23, p_{spin} = 0.006$
					-mPACC	$r = 0.66, p_{spin} < 0.001$	$r = 0.17, p_{spin} = 0.029$

bioRxiv preprint doi: https://doi.org/10.1101/2025.05.22.655469; this version posted May 25, 2025. The copyright holder for this preprint (which was not certified by peer review) is the author/funder, who has granted bioRxiv a license to display the preprint in perpetuity. It is made available under aCC-BY 4.0 International license.

Table 3: Gradient derivation method and FC thresholding has limited effect on the main results. This table shows the main analyses from Figure 2 and Figure 5 using gradients derived with principal component analysis (PCA) or diffusion map embedding (DME) over four different thresholding values: 0.00, 0.25, 0.50 and 0.75. A threshold of 0.75 means that 75% of the lowest values in the connectivity matrix is zeroed out. In the columns Gradient 1 and Gradient 3, the Pearson correlation (r) is shown with accompanying p-value from spin tests, quantifying the relationship between the t-values for each specific term and the Gradient scores based on the derivation method and thresholding specified in those two columns. Gradients were derived in all analyses from the average connectome of individuals without cognitive impairment, apoe4 non-carriers without abnormal amyloid levels. In BioFINDER only individuals ≤60 years of age were included while in ADNI the whole age range was used due to the smaller sample size. For the DME method this matrix is then converted into an affinity matrix by taking the cosine similarity between each pair of connectivity profiles, the components are then calculated as outlined in[35].

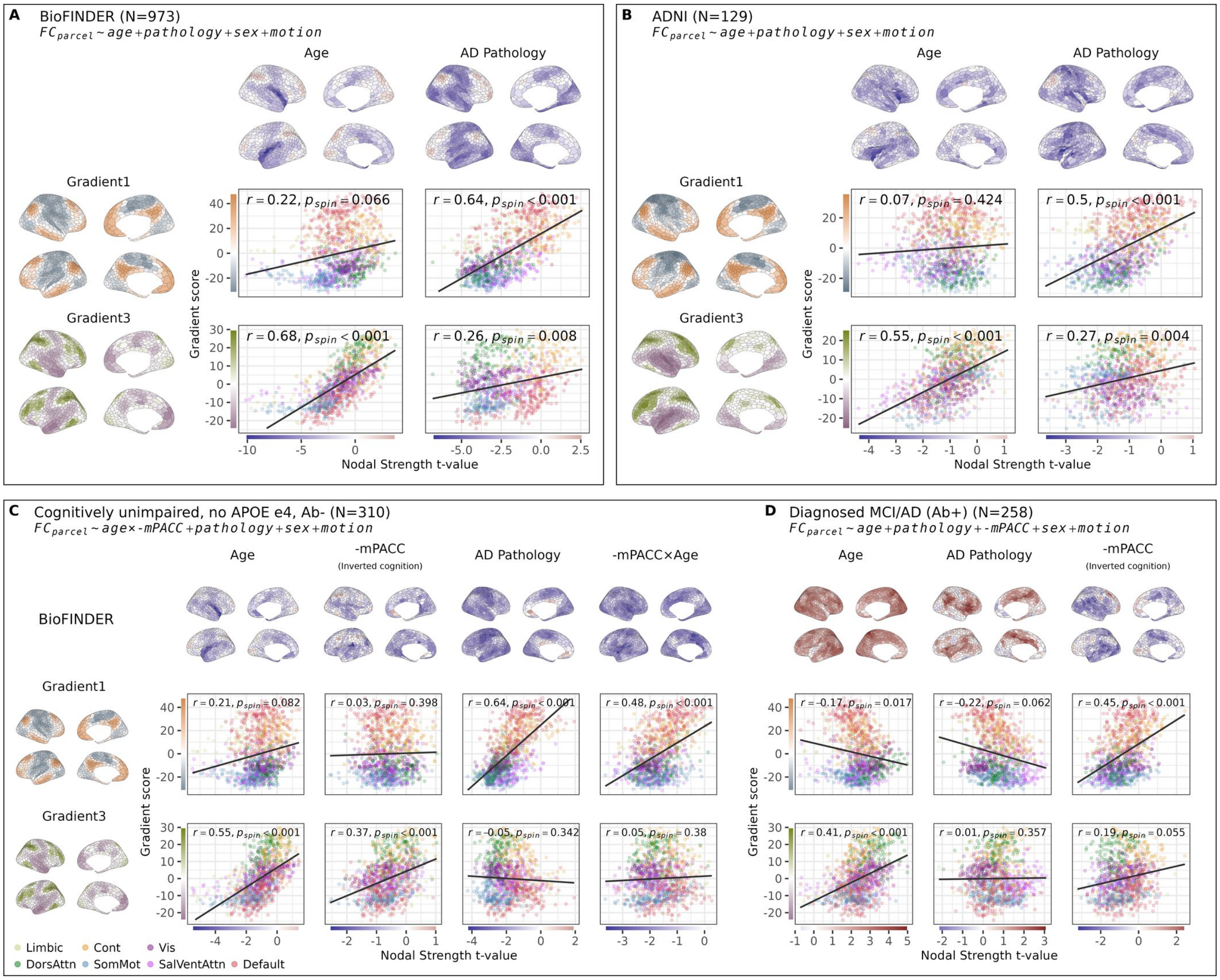

Figure S1: Analyses replicated with nodal connectivity strength (column-wise average of the unthresholded connectivity matrix) as the outcome measure demonstrates that age-related FC effects align with Gradient 3, while AD pathology-related effects align with Gradient 1, consistent with findings using nodal affinity in the main text. Results are shown for BioFINDER (A), ADNI (B). How cognitive status seems to modify these relationships are shown in (C) and (D). Cortical maps display t-values from nodal linear models, while scatter plots show the relationships between t-values and gradient scores, colored by net-

bioRxiv preprint doi: https://doi.org/10.1101/2025.05.22.655469; this version posted May 25, 2025. The copyright holder for this preprint (which was not certified by peer review) is the author/funder, who has granted bioRxiv a license to display the preprint in perpetuity. It is made available under aCC-BY 4.0 International license.

work membership. The relationship was quantified using Pearson correlation and significance assessed using a spin test.

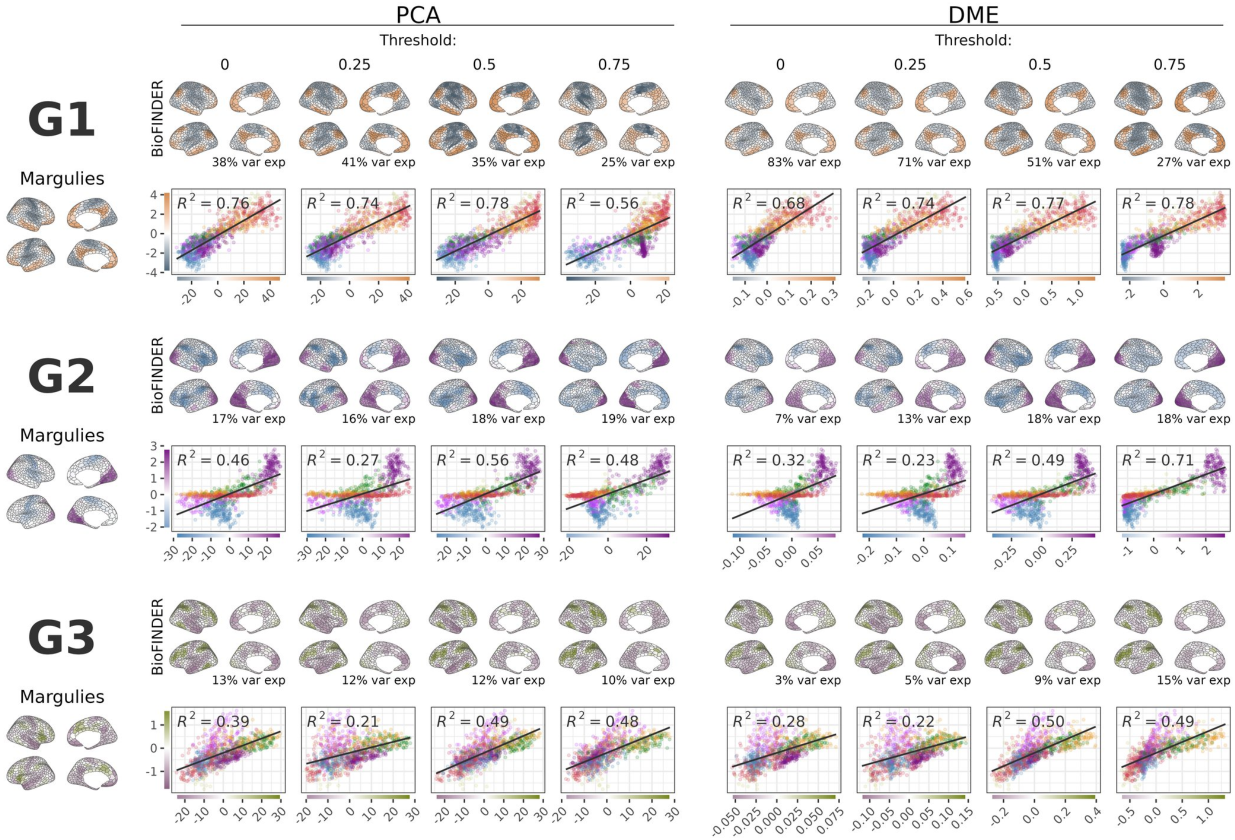

Figure S2: Derived gradients compared over a set of different parameter values show that the derivation method (PCA or DME) only has marginal effect on the overall spatial structure of the gradients. Reference gradients from[35] are presented in the leftmost column. PCA method: principal component analysis via singular value decomposition on the average connectivity matrix from individuals BioFINDER without cognitive impairment, APOE ε4 non-carriers without abnormal amyloid levels, and ≤60 years of age. DME: diffusion map embedding (DME). The gradients using either method are shown for 4 different levels of FC thresholding.

bioRxiv preprint doi: https://doi.org/10.1101/2025.05.22.655469; this version posted May 25, 2025. The copyright holder for this preprint (which was not certified by peer review) is the author/funder, who has granted bioRxiv a license to display the preprint in perpetuity. It is made available under aCC-BY 4.0 International license.

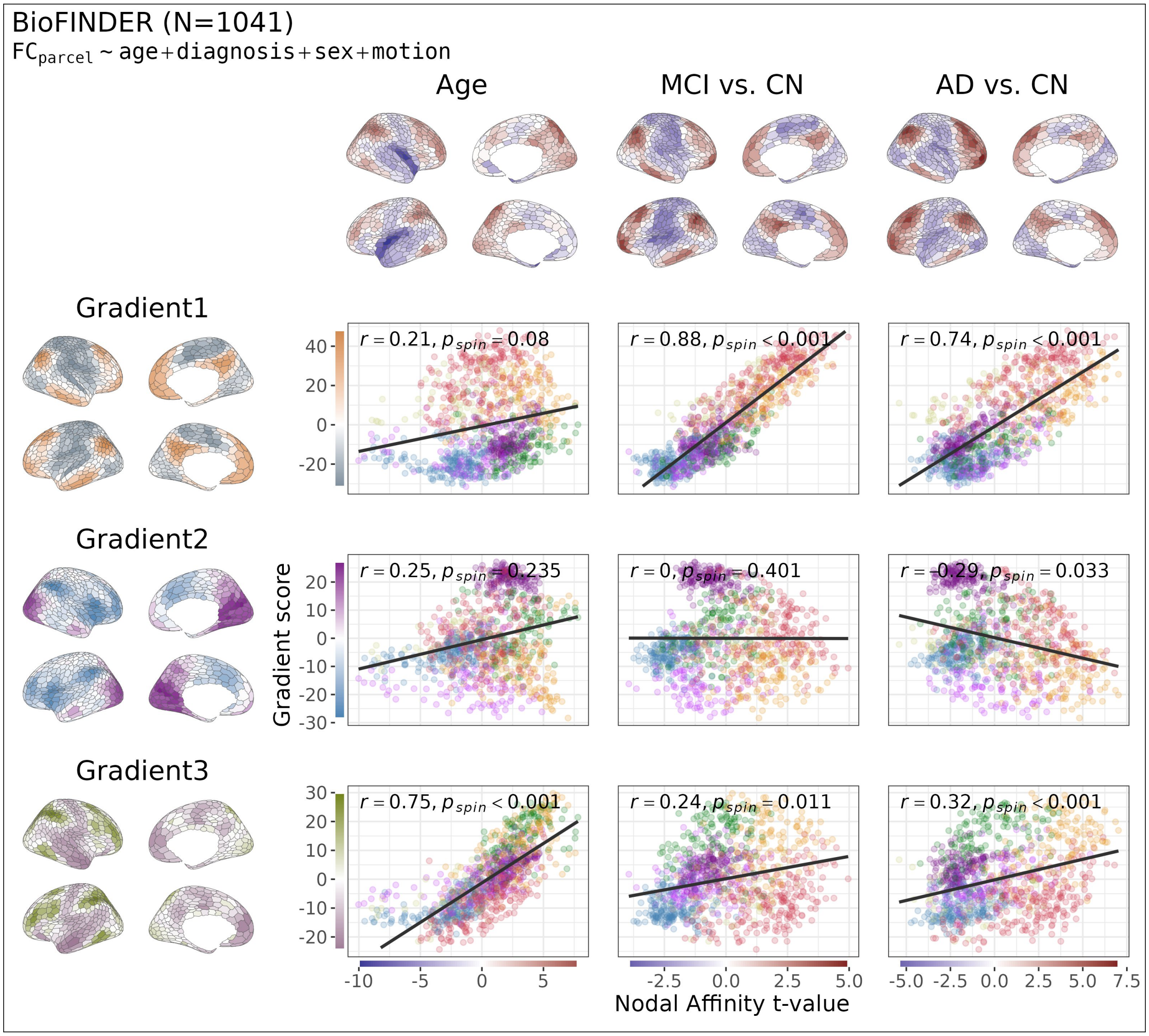

Figure S3: Analyses showing t-maps and alignment with organizational axes for age and clinical diagnoses. Cortical maps display t-values from nodal linear models, while scatter plots show the relationships between t-values and gradient scores, colored by network membership. The relationship was quantified using Pearson correlation and significance assessed using a spin test. The number of participants is here greater than in the main analysis due to more individuals having clinical diagnosis available than some of the pathology measures.

bioRxiv preprint doi: https://doi.org/10.1101/2025.05.22.655469; this version posted May 25, 2025. The copyright holder for this preprint (which was not certified by peer review) is the author/funder, who has granted bioRxiv a license to display the preprint in perpetuity. It is made available under aCC-BY 4.0 International license.

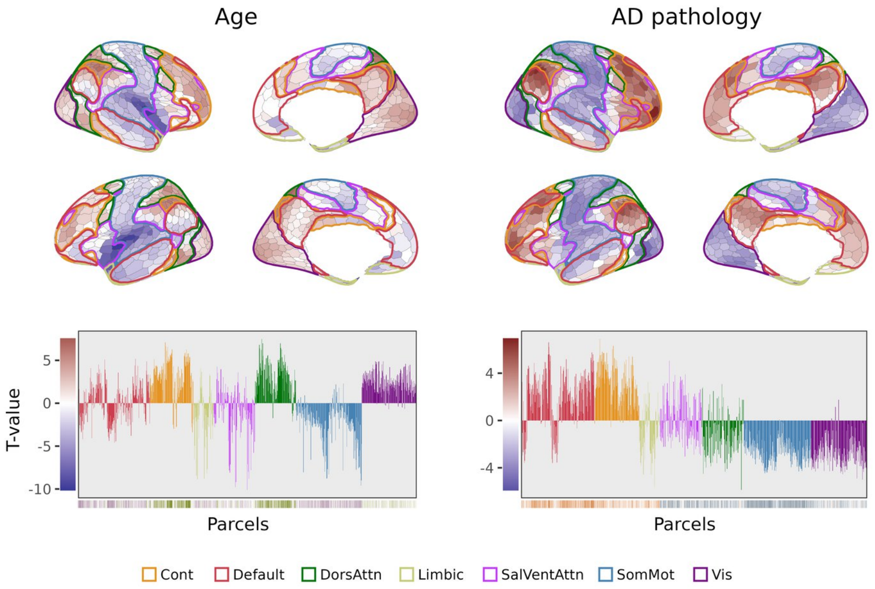

Figure S4: Main results of Figure 2 2A with the seven Yeo networks overlaid (top) and histograms of the t-values from all parcels colored by their network membership (bottom). The colorbar on the y-axes of the histograms correspond to the color scale of the cortical maps, and the colorbar on the x-axes correspond to the color scale of Gradient 3 and Gradient 1 for Age and AD pathology, respectively.

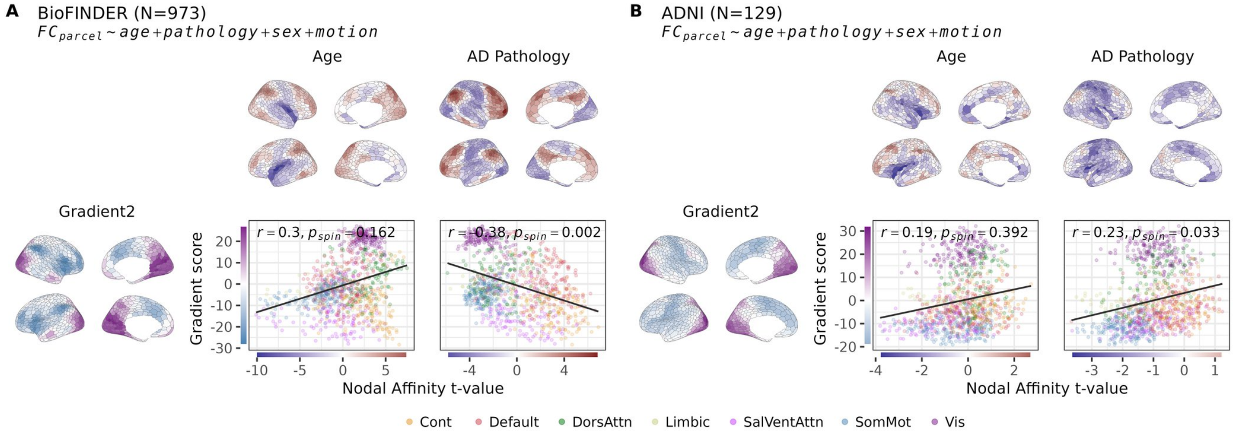

Figure S5: Main results of Figure 2 shown with Gradient 2. This analysis showed that there was no strong or reproducible relationships between the the t-maps for age and AD pathology and Gradient 2.

bioRxiv preprint doi: https://doi.org/10.1101/2025.05.22.655469; this version posted May 25, 2025. The copyright holder for this preprint (which was not certified by peer review) is the author/funder, who has granted bioRxiv a license to display the preprint in perpetuity. It is made available under aCC-BY 4.0 International license.

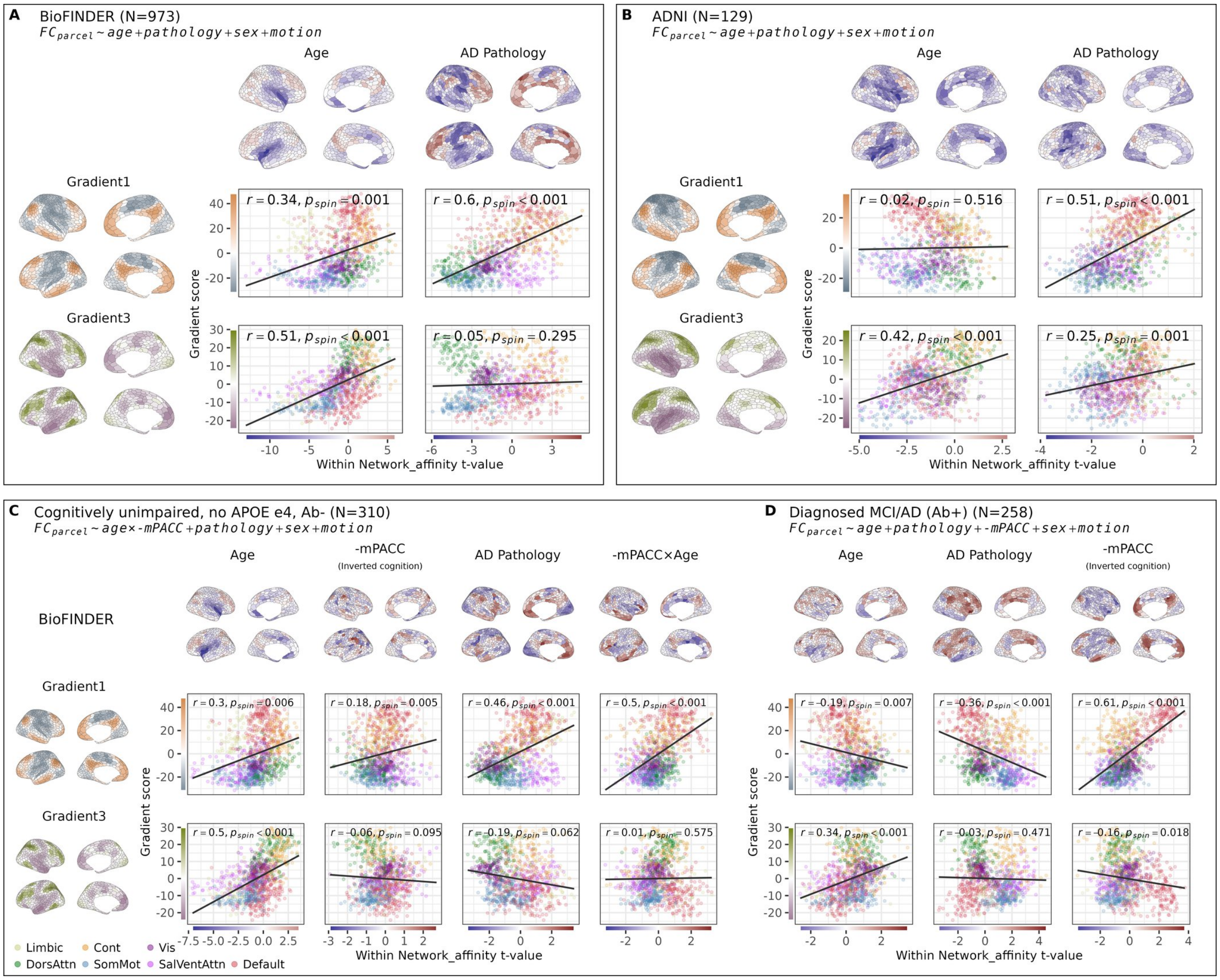

Figure S6: Analyses replicated with within-network affinity. Within-network affinity was calculated as the average affinity between parcels within the same Yeo network[113]. Results are shown for BioFINDER (A), ADNI (B). How cognitive status seems to modify these relationships are shown in (C) and (D). Cortical maps display t-values from nodal linear models, while scatter plots show the relationships between t-values and gradient scores, colored by network membership. The relationship was quantified using Pearson correlation and significance assessed using a spin test.

bioRxiv preprint doi: https://doi.org/10.1101/2025.05.22.655469; this version posted May 25, 2025. The copyright holder for this preprint (which was not certified by peer review) is the author/funder, who has granted bioRxiv a license to display the preprint in perpetuity. It is made available under aCC-BY 4.0 International license.

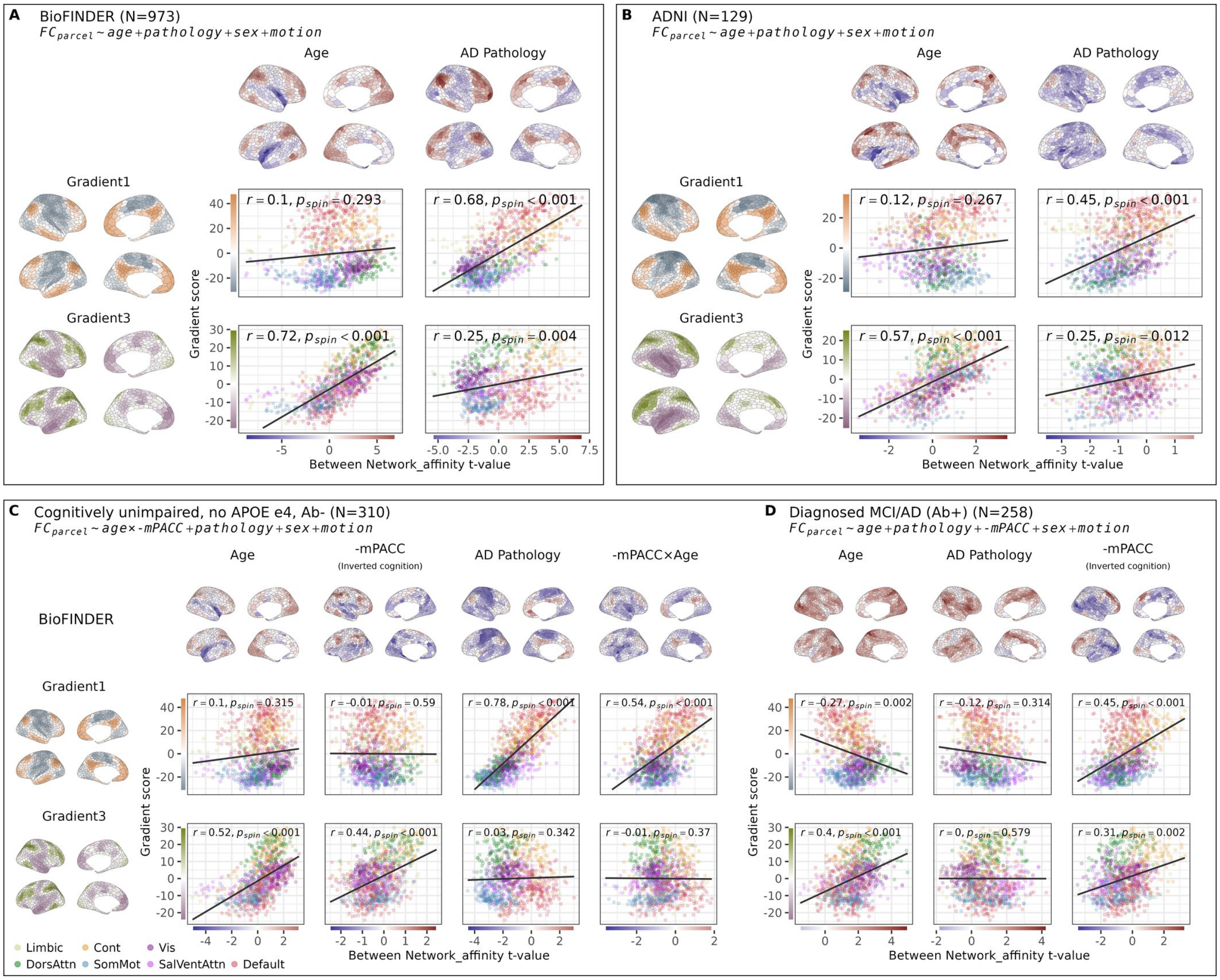

Figure S7: Analyses replicated with between-network affinity. Between-network affinity was calculated as the average affinity to parcels outside of a parcel's own Yeo 7 network[113]. Results are shown for BioFINDER (A), ADNI (B). How cognitive status seems to modify these relationships are shown in (C) and (D). Cortical maps display t-values from nodal linear models, while scatter plots show the relationships between t-values and gradient scores, colored by network membership. The relationship was quantified using Pearson correlation and significance assessed using a spin test.

bioRxiv preprint doi: https://doi.org/10.1101/2025.05.22.655469; this version posted May 25, 2025. The copyright holder for this preprint (which was not certified by peer review) is the author/funder, who has granted bioRxiv a license to display the preprint in perpetuity. It is made available under aCC-BY 4.0 International license.

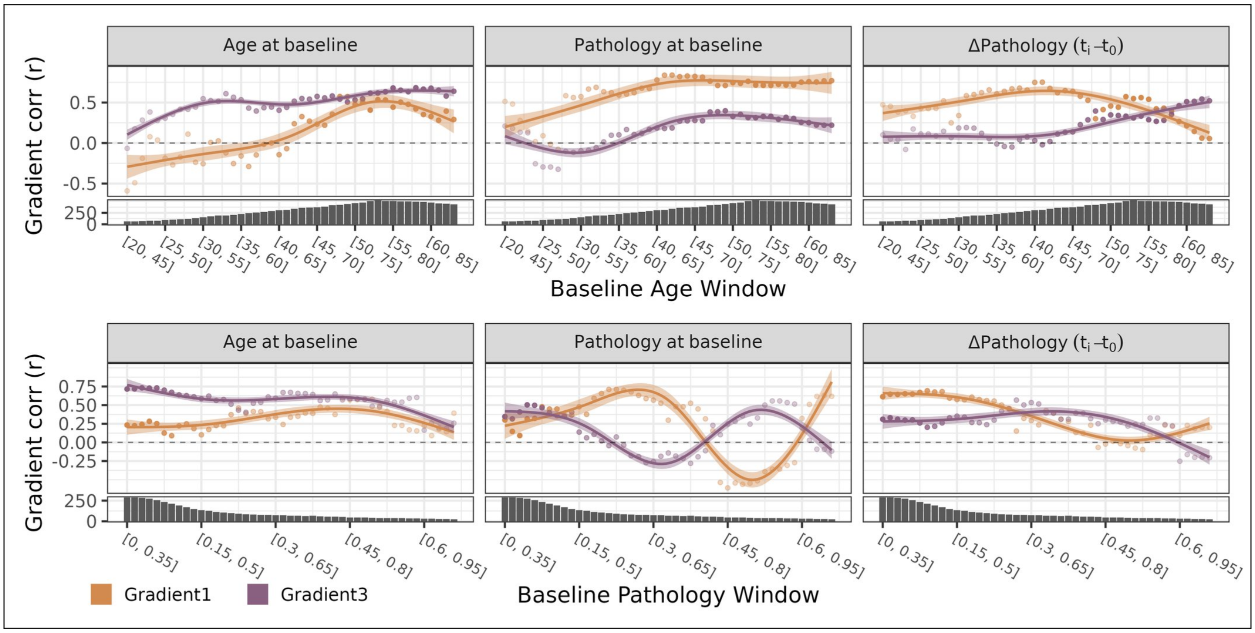

Figure S8: Results of the sliding window analysis from Figure 4 showing the dynamic effect of AD pathology on FC gradient alignment. The correlation values on the scatters are smoothed with generalized additive models. Correlations are shown for each term for both Gradient 1 and 3. Marginal plots show the sample size for each window.

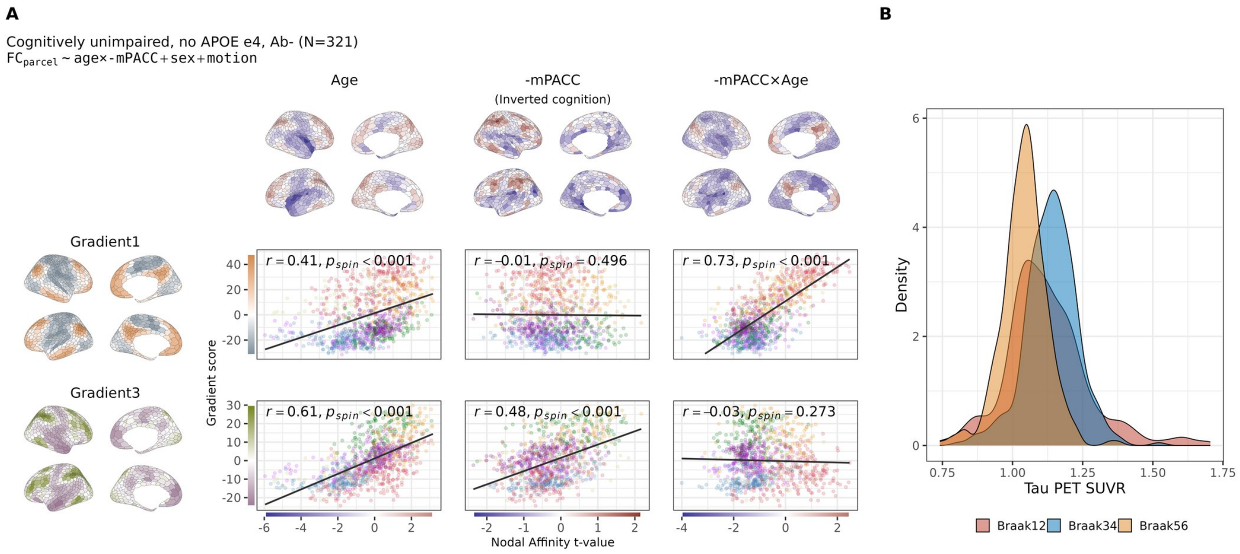

Figure S9: A: Cross-sectional results for cognitively unimpaired Aβ-, APOE ε4 non-carriers. The model includes age, inverted cognition (-mPACC), and an age × cognition interaction (with centered predictors and inverted cognition scores for interpretability). Unlike the main analysis presented in Figure 5, this analysis does not adjust for pathology. The age effects observed here partially reflect the effects previously attributed to pathology, revealing a relationship with Gradient 1 that diminishes when adjusting for pathology. Cortical maps display t-values from nodal linear regression models, while scatter plots

bioRxiv preprint doi: https://doi.org/10.1101/2025.05.22.655469; this version posted May 25, 2025. The copyright holder for this preprint (which was not certified by peer review) is the author/funder, who has granted bioRxiv a license to display the preprint in perpetuity. It is made available under aCC-BY 4.0 International license.

show the relationships between the t-values and gradient scores, colored by network membership. The relationship was quantified using Pearson correlation and significance assessed using a spin test. B: Distribution of tau PET SUVR from Braak I-II, Braak III-IV and Braak V-VI in cognitively unimpaired Aβ-, APOE ε4 non-carriers.

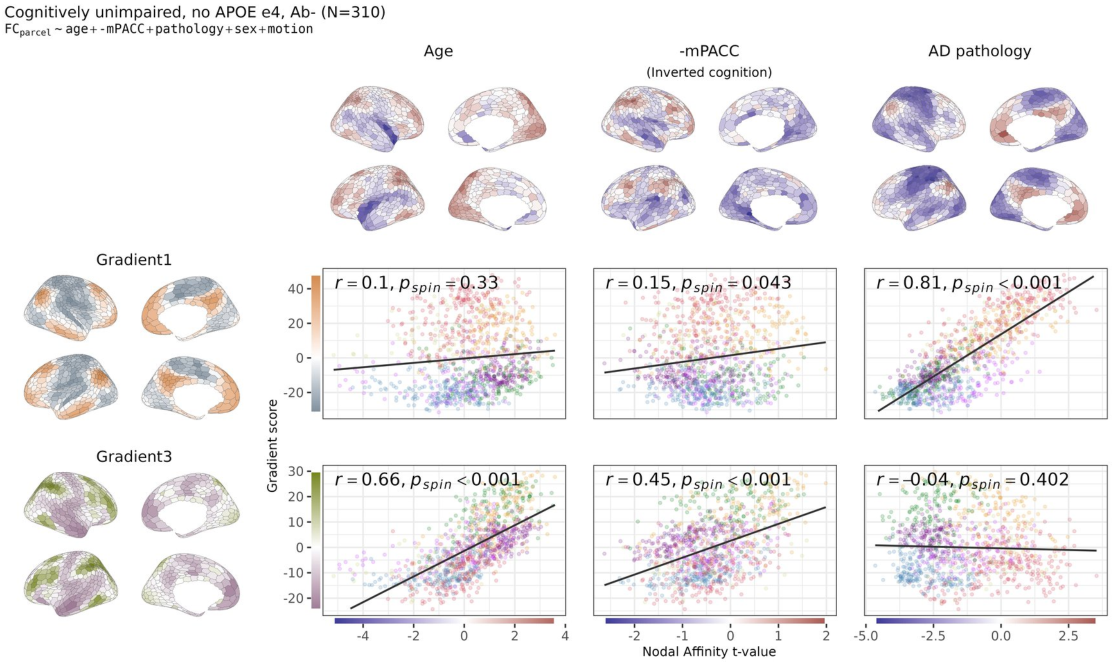

Figure S10: Cross-sectional results for cognitively unimpaired Aβ-, APOE ε4 non-carriers. This model is without interaction between cognition and age.

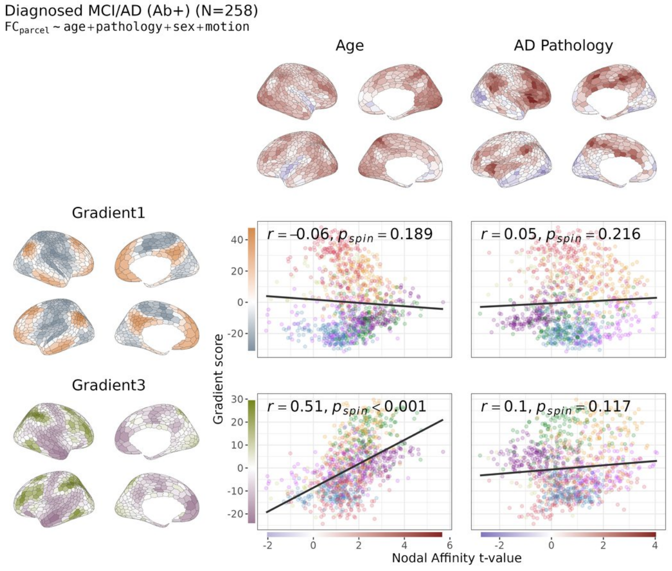

bioRxiv preprint doi: https://doi.org/10.1101/2025.05.22.655469; this version posted May 25, 2025. The copyright holder for this preprint (which was not certified by peer review) is the author/funder, who has granted bioRxiv a license to display the preprint in perpetuity. It is made available under aCC-BY 4.0 International license.

Figure S11: Cross-sectional results within the MCI and AD group without cognition. This analysis revealed that AD pathology showed no relationship to Gradient 1 even when not accounting for cognition, within this group.

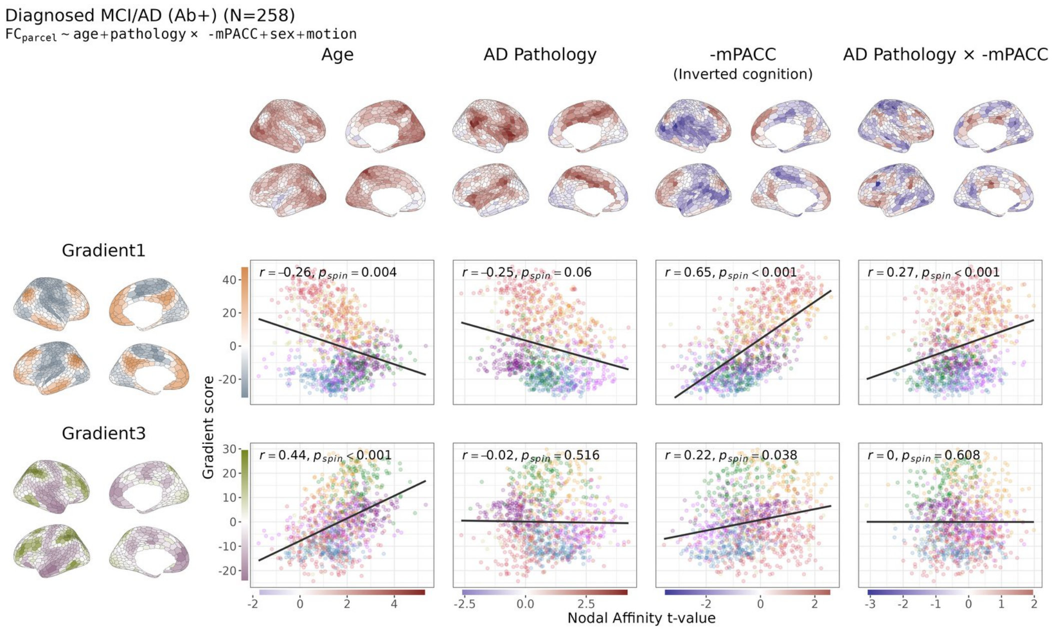

Figure S12: Cross-sectional analyses within patients with mild cognitive impairment (MCI) and Alzheimer's disease (AD), interacting AD pathology and cognition revealed that this did not affect the main effect of cognition. Predictors have been centred and cognition scores inverted for interpretability. Cortical maps display t-values from nodal linear regression models, while scatter plots show the relationships between the t-values and gradient scores, colored by network membership. The relationship was quantified using Pearson correlation and significance assessed using a spin test.

bioRxiv preprint doi: https://doi.org/10.1101/2025.05.22.655469; this version posted May 25, 2025. The copyright holder for this preprint (which was not certified by peer review) is the author/funder, who has granted bioRxiv a license to display the preprint in perpetuity. It is made available under aCC-BY 4.0 International license.

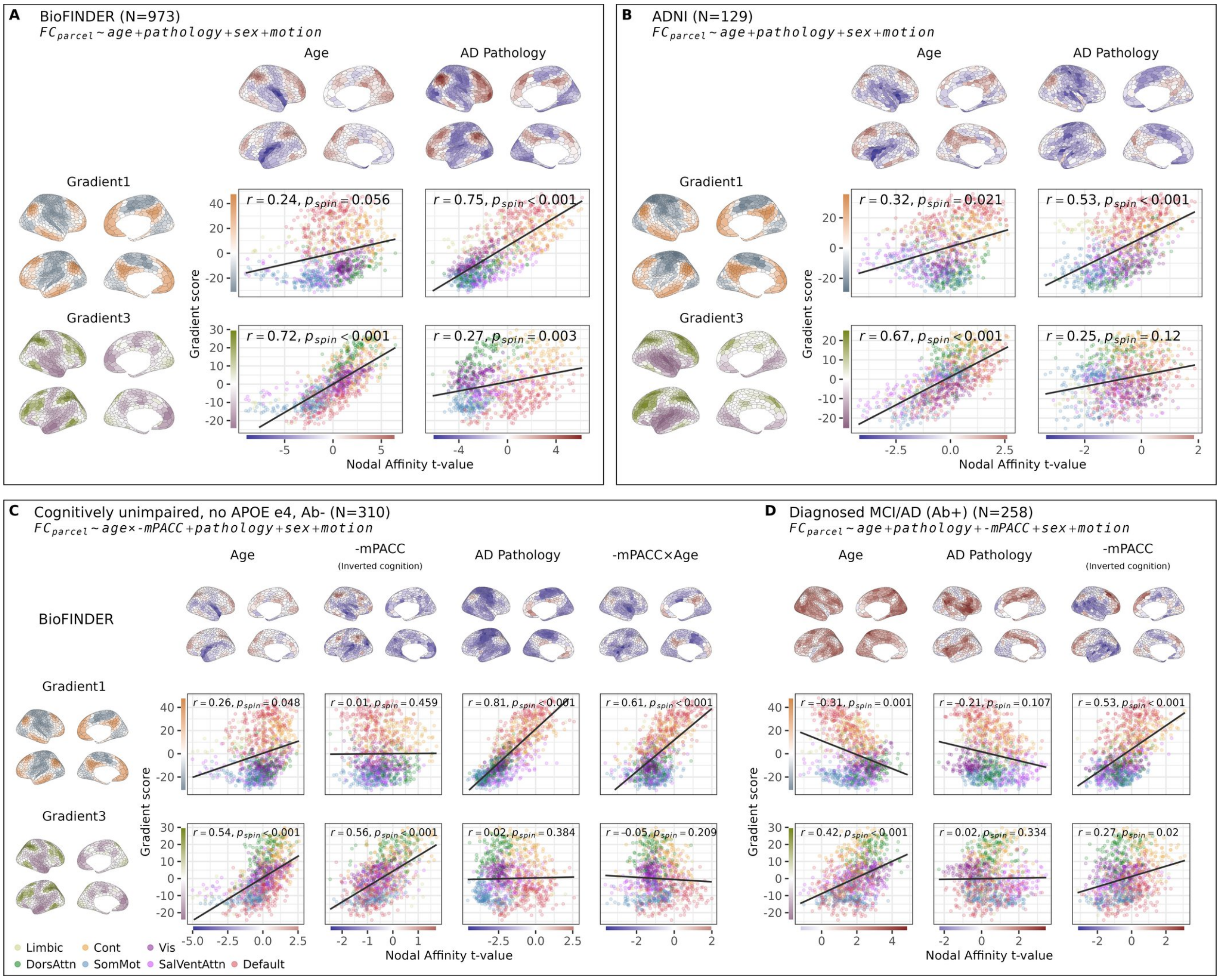

Figure S13: Analyses replicated with nodal affinity calculated using Pearson correlation instead of cosine similarity and no thresholding demonstrate consistent results with the main analyses (Figure 2 and Figure 5). Results are shown for BioFINDER (A), ADNI (B). How cognitive status seems to modify these relationships are shown in (C) and (D). Cortical maps display t-values from nodal linear models, while scatter plots show the relationships between t-values and gradient scores, colored by network membership. The relationship was quantified using Pearson correlation and significance assessed using a spin test.
